# Altered levels of CSF proteins in patients with FTD, presymptomatic mutation carriers and non-carriers

**DOI:** 10.1186/s40035-020-00198-y

**Published:** 2020-06-23

**Authors:** Julia Remnestål, Linn Öijerstedt, Abbe Ullgren, Jennie Olofsson, Sofia Bergström, Kim Kultima, Martin Ingelsson, Lena Kilander, Mathias Uhlén, Anna Månberg, Caroline Graff, Peter Nilsson

**Affiliations:** 1 Division of Affinity Proteomics, Department of Protein Science, KTH Royal Institute of Technology, SciLifeLab, Tomtebodavägen 23 A, Alpha 2, 171 65 Solna, Stockholm, Sweden; 2 Swedish FTD Initiative, Stockholm, Sweden; 3 Division of Neurogeriatrics, Centre for Alzheimer Research, Department of Neurobiology, Care Sciences and Society, Karolinska Institutet, 171 64 Solna, Sweden; 4 Unit for hereditary dementias, Theme Aging, Karolinska University Hospital, Stockholm, Sweden; 5 Department of Medical Sciences, Clinical Chemistry, Uppsala University, Uppsala, Sweden; 6 Department of Public Health and Caring Sciences, Geriatrics, Uppsala University, Uppsala, Sweden; 7 Department of Neuroscience, Karolinska Institutet, Solna, Sweden

**Keywords:** Frontotemporal dementia, Cerebrospinal fluid, Biomarkers, Proteomics, Antibody suspension bead array

## Abstract

**Background:**

The clinical presentations of frontotemporal dementia (FTD) are diverse and overlap with other neurological disorders. There are, as of today, no biomarkers in clinical practice for diagnosing the disorders. Here, we aimed to find protein markers in cerebrospinal fluid (CSF) from patients with FTD, presymptomatic mutation carriers and non-carriers.

**Methods:**

Antibody suspension bead arrays were used to analyse 328 proteins in CSF from patients with behavioural variant FTD (bvFTD, *n* = 16) and progressive primary aphasia (PPA, *n* = 13), as well as presymptomatic mutation carriers (PMC, *n* = 16) and non-carriers (NC, *n* = 8). A total of 492 antibodies were used to measure protein levels by direct labelling of the CSF samples. The findings were further examined in an independent cohort including 13 FTD patients, 79 patients with Alzheimer’s disease and 18 healthy controls.

**Results:**

We found significantly altered protein levels in CSF from FTD patients compared to unaffected individuals (PMC and NC) for 26 proteins. The analysis show patterns of separation between unaffected individuals and FTD patients, especially for those with a clinical diagnosis of bvFTD. The most statistically significant differences in protein levels were found for VGF, TN-R, NPTXR, TMEM132D, PDYN and NF-M. Patients with FTD were found to have higher levels of TN-R and NF-M, and lower levels of VGF, NPTXR, TMEM132D and PDYN, compared to unaffected individuals. The main findings were reproduced in the independent cohort.

**Conclusion:**

In this pilot study, we show a separation of FTD patients from unaffected individuals based on protein levels in CSF. Further investigation is required to explore the CSF profiles in larger cohorts, but the results presented here has the potential to enable future clinical utilization of these potential biomarkers within FTD.

## Background

Frontotemporal dementia (FTD) is one of the most common forms of early onset dementia. The clinical presentations are diverse and overlap with other psychiatric and neurological disorders [1]. The major phenotypes are behavioural variant FTD (bvFTD) and primary progressive aphasia (PPA). Patients with bvFTD present personality changes such as inappropriate behaviour, impaired judgement and loss of empathy. PPA can be further divided into progressive non-fluent aphasia (PNFA), in which patients have effortful speech with phonetic errors, and semantic dementia (SD) in which patients have fluent speech but impaired single-word comprehension [1, 2]. In addition, approximately 15% of FTD patients develop amyotrophic lateral sclerosis (ALS) and other motoric symptoms [3]. This phenotypic heterogeneity and lack of objective diagnostic tests may result in misdiagnosis and incorrect care of patients and their families [4, 5].

A family history of dementia is found in up to half of patients with FTD [6]. Several genetic variants are known to cause FTD, and are most frequently identified in the genes *C9orf72*, progranulin (*GRN*) and ﻿microtubule-associated protein tau (*MAPT*) [7–11]. In Sweden, the frequency of the *C9orf72* repeat expansion mutation is particularly high [12, 13]. The pathogenic mutations are weakly associated with the different FTD phenotypes [14]. Genetic testing is useful for diagnostic confirmation of a genetic cause of FTD but has limited value for prognosis of age at onset or phenotype in presymptomatic individuals. Numerous studies have investigated potential protein biomarkers for FTD in cerebrospinal fluid (CSF) [15–18] and one promising candidate is neurofilament light chain (NF-L). In 2016, Meeter et al. showed that CSF NF-L is elevated in patients with FTD compared to controls and suggested that the levels correlate to disease severity [16, 19]. However, elevated levels of CSF NF-L have also been found in several other neurodegenerative diseases and are thus not specific for FTD [20, 21].

This far, most studies have focused on analysing single candidate proteins. Here, we utilize antibody based suspension bead arrays that enabled a high throughput multiplex screening of 328 proteins in 53 CSF samples from FTD patients, presymptomatic mutation carriers and non-carriers from Sweden. We aimed to find differences in protein profiles between patients, presymptomatic mutation carriers and non-carriers to discover novel protein biomarkers for further clinical validation.

## Methods

### Participants

The exploratory cohort 1 was recruited at the Memory clinic at Karolinska University Hospital and consisted of both patients diagnosed with FTD and unaffected subjects enrolled in the GENFI-study (GENetic Frontotemporal Dementia Initiative): 29 were diagnosed with FTD, between 1997 and 2016, according to the criteria by Rascovsky et al. 2008 or Gorno-Tempini et al 2011 [1, 2] and 24 were clinically unaffected participants from the GENFI-study (Table 1) [22, 23]. All the unaffected participants were at 50% risk of FTD due to a confirmed pathogenic mutation in a first degree relative. Throughout the text, “unaffected individuals” will be used as a collective term for presymptomatic mutation carriers and non-carriers. The study was approved by the Regional Ethical Review Board, Stockholm, Sweden (registration numbers: 2017/834–31/1, 2012/1611–31/3, 2013/1563–32, 2017/2097–32).

**Table 1:** Cohort demographics

**Cohort 1**				
	**NC** (*n* = 8)	**PMC** (*n* = 16)	**PPA** (*n* = 13)	**bvFTD** (*n* = 16)
Age, median years (range)^a^	52 (24–65)	53 (31–71)	65 (52–79)	61 (40–78)
Female, N (%)	4 (50)	10 (62)	8 (57)	6 (37)
Age at onset, median years (range)	–	–	63 (50–78)	59 (39–77)
Years to expected onset^b^, median years (range)	–	8 (−24,+ 3)	–	–
Mutation, N (%)				
*C9orf72*	–	8 (50)	0	2 (13)
*GRN*	–	8 (50)	1 (8)	–
*VCP*	–	–	–	1 (6)
**Cohort 2**	**Control** (*n* = 18)	**FTD**^c^ (*n* = 13)	**AD** (*n* = 79)	
Age, median years (range)^d^	81 (74–86)	68 (50–83)	72 (54–88)	
Female, N (%)	10 (56)	4 (31)	49 (62)	

*NC* Non carriers, *PMC* Presymptomatic mutation carriers, *PPA* Primary progressive aphasia, bvFTD – behavioural variant FTD, *AD* Alzheimer’s disease

^a^ Differences in age were found between PPA and unaffected individuals (ANOVA, *p* = 0.001, pairwise post hoc test PPA vs NC, *p* = 0.01, PPA vs PMC, *p* = 0.01). Differences where found between FTD as a whole group and unaffected individuals (t-test, *p* < 0.001)

^b^ Difference between the subjects age at sampling and the mean age at onset in their family

^c^ Clinical phenotype: 7 bvFTD and 5 SD

^d^ Differences in age were found between controls and FTD/AD (ANOVA, *p* < 0.001, pairwise post hoc test controls vs FTD, *p* < 0.001, controls vs AD, *p* < 0.001)

An independent cohort from Uppsala University Hospital (cohort 2) consisting of 13 patients diagnosed with FTD, 79 patients diagnosed with Alzheimer’s disease (AD) and 18 healthy individuals were used to replicate the main findings in the cohort collected at the Karolinska University Hospital Memory clinic (Table 1). The study was approved by the Regional Ethical Review Board in Uppsala, Sweden (registration numbers: 2005–244, Ö 48–2005; 2005-11-02, 2006-01-30, 2011/044; 2011-02-23).

All participants gave informed consent to research, including DNA and CSF sampling. Detailed information about patient recruitment and sample collection can be found in Supplementary Materials and Methods.

### Genetic screening

FTD patients recruited at the Karolinska University Hospital Memory clinic were screened for mutations in *C9orf72, GRN* and *MAPT* (*n* = 25) or by whole genome sequencing (*n* = 4) (see Supplementary Materials and Methods). Unaffected subjects were screened for the mutation segregating in the family using Sanger-sequencing (for *MAPT* and *GRN*) or repeat primed PCR (for *C9orf72* repeat expansion). Thus, the mutation status was known in cohort 1.

### Protein profiling

Antibody suspension bead arrays were used to explore the protein profiles in human CSF. A total of 328 proteins, targeted by 492 antibodies, were included in the experimental analysis. Creation of the suspension bead array was done by immobilizing antibodies onto magnetic, colour coded carboxylated beads as described previously [24–27]. Fifteen μl of each CSF sample was diluted, labelled with biotin and detection was enabled by a streptavidin coupled fluorophore, reported as median fluorescence intensity (MFI) from at least 30 beads per bead identity and sample (see Supplementary Materials and Methods).

### Validation of NF-M antibodies

To ensure that the antibodies HPA023138 and HPA022845 captured neurofilament medium polypeptide (NF-M) in the studied sample material, a sandwich assay directed toward NF-M was developed according to a previously published workflow [28] (see Supplementary Materials and Methods).

### Data processing and statistical analysis

All data analysis and data visualizations were performed using the open source software R version 3.5.1 [29].

#### Demographics

Tests of normality were performed using Shapiro-Wilk test (age, age at onset, years to expected onset). ANOVA with Bonferroni post hoc tests were used for assessing differences in age between patients and unaffected individuals. Due to a violation of the assumption of normality, Mann-Whitney U test was used when assessing the variable age at onset. When assessing sex differences, Fischer’s exact test was used as the expected values was < 5 in more than 20% of the contingency cells. *P*-values of < 0.05 were considered significant.

#### Analysis of protein profiles

Differences in protein levels between patients with FTD and unaffected individuals were investigated using principal component analysis (PCA) from a set of antibodies with intra-assay coefficient of variance (CV) < 10% and inter-assay rho > 0.8. To avoid bias from multiple antibodies targeting the same protein, one antibody was selected for each target. All protein values were univariance scaled and centered as in R function prcomp default. The antibody selection for the PCA analysis was made based on the highest degree of technical validation within the Human protein atlas project (HPA, www.proteinatlas.org). All group comparisons were made using the Mann-Whitney U test and all *p*-values were false discovery rate (FDR) adjusted by the Benjamini-Hochberg procedure for multiple comparisons [30]. Adjusted p-values of < 0.01 were considered significant. Generalized linear models for gamma distributed variables were performed on log transformed data to examine effects of age at sampling on protein level differences between the studied groups. Two hierarchical clustering models were made using complete linkage. The first one using the 10 first principal components from the PCA and the second one using intensity levels of a selected set of proteins. The clustering was presented as dendrograms using a cut off of four clusters, based on the number of subject groups. All additional protein or antibody correlations were calculated using Spearman’s rho statistics.

## Results

### Demographics

Cohort 1 consisted of 29 patients diagnosed with FTD and 24 unaffected individuals with a 50% risk of genetic FTD. Of the 29 FTD patients, sixteen fulfilled criteria for bvFTD, eight for PNFA and four for SD. Four of the patients displayed symptoms of motor neuron disease. One patient with PPA could not be subclassified as neither PNFA nor SD. This individual had anomic aphasia and alexia with agraphia but did not fulfil criteria for SD. Magnetic resonance imaging showed atrophy of the left temporal cortex. CSF total-tau (291 ng/L), phospho-tau (50 ng/L) and Amyloid beta 42 (489 ng/L) were within normal range. Four of the FTD patients were found to have a pathogenic mutation, two with the *C9orf72* repeat expansion mutation, one with a *GRN* mutation and one with a *valosin containing protein* (*VCP*) mutation.

Among the 24 unaffected individuals, 8 were non-mutation carriers (NC), 8 were *C9orf72* mutation carriers and 8 were *GRN* mutation carriers. These mutation carriers are denoted as presymptomatic mutation carriers, (PMC) (Table 1). When comparing the mean age at sampling in the four groups (NC, PMC, bvFTD and PPA), differences were only found between PPA and unaffected (PPA, 65 years; PMC, 53 years; NC, 52 years; ANOVA, *p* = 0.001, pairwise post hoc test PPA vs NC, *p* = 0.01 and PPA vs PMC, *p* = 0.01). Differences in age were found between FTD as a whole group and unaffected individuals (FTD, 64 years; unaffected, 53 years; t-test, *p* < 0.001). The sex distribution was not statistically different between the four groups (Fisher’s exact test, *p* = 0.49). Patients with PPA had a later age at onset compared to bvFTD (mean 63 vs 59 years).

Cohort 2 consisted of 13 patients diagnosed with FTD (7 with bvFTD and 5 with SD), 79 patients diagnosed with AD and 18 healthy individuals. When comparing the mean age at sampling in the three groups, differences were found between controls and FTD/AD (ANOVA, *p* < 0.001, pairwise post hoc test controls vs FTD, *p* < 0.001, controls vs AD, *p* < 0.001) (Table 1).

### Analysis of protein profiles

**Fig. 1:**
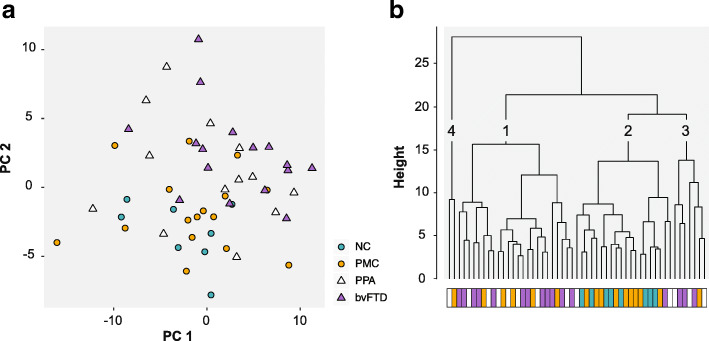
PCA and cluster dendrogram. **a** PCA plot of principal component 1 and 2. Unaffected individuals are shown as circles and FTD patients as triangles. **b** Cluster dendrogram of principal component 1 to 10. The four groups are indicated by the coloured bar and numbers have been added to each cluster for clarification. NC – Non-carriers, PMC – Presymptomatic mutation carriers, PPA – Primary progressive aphasia, bvFTD – Behavioural variant FTD

A principal component analysis of 70 proteins (targeted by the most robust antibodies, which fulfilled intra-assay CV < 10% and inter-assay rho> 0.8) showed that the majority of the distribution of differences in protein levels could be explained by principal components 1 to 10 (90%). Figure 1a illustrates differences in protein levels between patients with FTD, PMC and NC. Unaffected individuals mainly cluster at the bottom left and bvFTD patients at the upper right while PPA patients are scattered around the plot.

Differences between all FTD patients (bvFTD and PPA) and unaffected individuals were further visualized by hierarchical clustering of PC 1 to 10. Two major clusters were revealed (Fig. 1b): one including 12/16 (75%) of the bvFTD patients and 7/13 (54%) of the PPA patients (Cluster 1), and the other including all NC and the majority (56%) of PMC (Cluster 2). A third minor cluster was also observed consisting of three PPA patients, three bvFTD patients and a single PMC (Cluster 3). Two individuals (one PMC and one PPA) made one separate cluster (Cluster 4).

### Protein level differences between bvFTD, PPA, PMC and NC

**Fig. 2:**
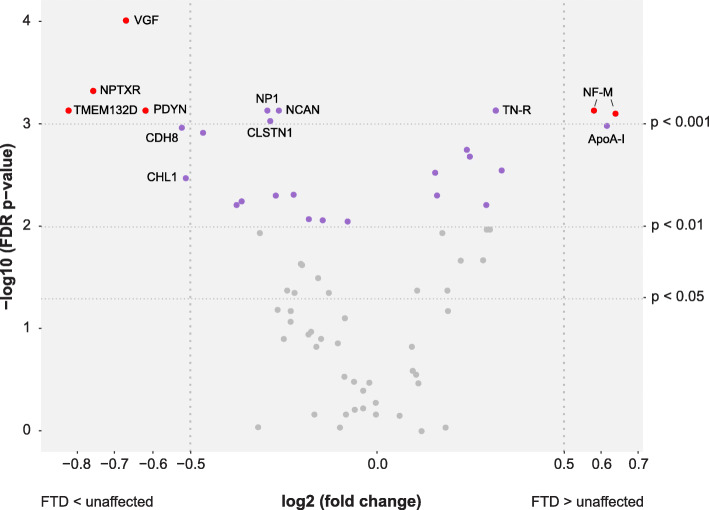
Volcano plot of analysed proteins (*n* = 70). Differences in protein levels between FTD patients and unaffected individuals displayed by log2(fold change) and significance level displayed as -log10(p). All proteins with significant differences (FDR adjusted *p* < 0.01) are displayed in purple. Proteins highlighted with gene names have a *p* < 0.001 or an absolute log2(fold change) > 0.5 (corresponding to a fold change < 0.7 or > 1.4). The five proteins with both a *p* < 0.001 and an absolute log2(fold change) > 0.5 are displayed in red

**Fig. 3:**
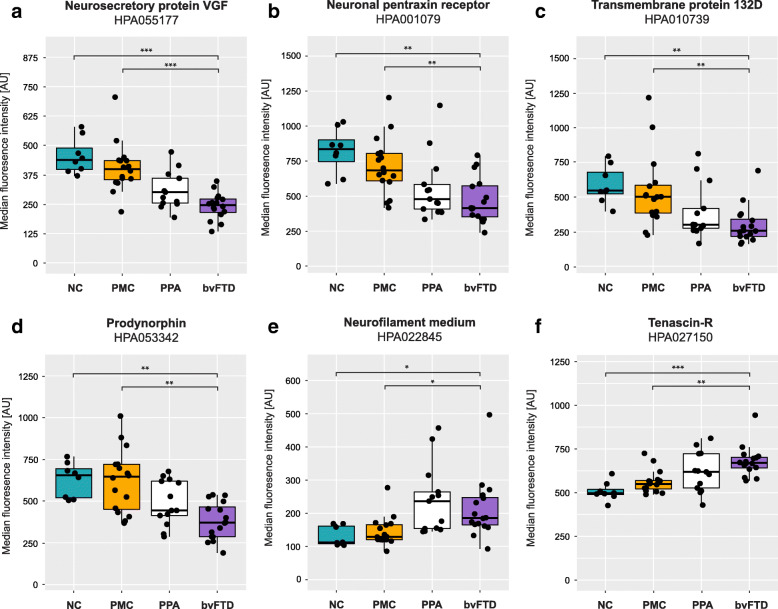
CSF levels in the different clinical subgroups (NC, PMC, PPA and bvFTD). (**a**) neurosecretory protein VGF (VGF), (**b**) neuronal pentraxin receptor (NPTXR), (**c**) transmembrane protein 132D (TMEM132D), (**d**) prodynorphin (PDYN), (**e**) neurofilament medium polypeptide (NF-M) and (**f**) tenascin-R (TN-R). Stars indicate significant differences, * *p* < 0.05, ** *p* < 0.01 and ***p < 0.001 NC – Non-carriers, PMC – Presymptomatic mutation carriers, PPA – Primary progressive aphasia, bvFTD – Behavioural variant FTD

The PCA indicated differences in protein profiles between FTD patients and unaffected individuals. Comparisons were also made for each protein separately (*n* = 70), illustrated in Fig. 2 and Supplementary Table 1. Statistically significant differences in protein levels (FDR adjusted *p* < 0.01) between FTD patients and unaffected individuals were found for 26 proteins (purple and red points). Nine of the 26 proteins showed differences with an FDR adjusted *p*-value below 0.001: neurosecretory protein VGF (VGF), neuronal pentraxin receptor (NPTXR), transmembrane protein 132D (TMEM132D), prodynorphin (PDYN), neurofilament medium polypeptide (NF-M) (with two independent antibodies), tenascin-R (TN-R), neuronal pentraxin-1 (NP1), neurocan core protein (NCAN) and calsyntenin-1 (CLSTN1). An absolute log2 fold change larger than 0.5 (corresponding to a fold change < 0.7 or > 1.4) was observed for eight proteins: TMEM132D, NPTXR, VGF, NF-M, PDYN, apolipoprotein A-I (ApoA-I), cadherin-8 (CDH8) and neural cell adhesion molecule L1-like protein (CHL1). The five proteins VGF, NPTXR, TMEM132D, PDYN and NF-M showed both an absolute fold change > 0.5 and statistically significant differences (FDR adjusted *p* < 0.001) (Fig. 2, red points, Fig. 3a-e). Nine of the 26 proteins were elevated in FTD patients compared to unaffected individuals, while lower levels were observed for the other 17 proteins. Generalized linear models showed that age had a significant effect (*p* < 0.05) on the differences between the two groups for eight proteins; CLSTN1, Peptidyl-glycine alpha-amidating monooxygenase, ApoA-I, Leucine-rich alpha-2-glycoprotein (LRG), Alpha-1-antichymotrypsin (ACT), Inter-alpha-trypsin inhibitor heavy chain H1, Oligodendrocyte-myelin glycoprotein and Phosphoinositide-3-kinase-interacting protein 1 (Table 2 and Supplementary Table 2).

**Table 2:** List of proteins with significant differences in protein profiles. Sorted by *p*-values for comparison of bvFTD and NC (lowest to highest)

Protein name	Short name	Uniprot ID	Antibody	bvFTD vs NC	bvFTD vs PMC	FTD vs unaffected	Direction of change^**c**^
*Neurosecretory protein VGF*	VGF	O15240	HPA055177	0.0002	0.0003	0.0001^b^	↓
*Tenascin-R*	TN-R	Q92752	HPA027150	0.0008	0.002	0.0007^b^	↑
*Neuronal pentraxin receptor*	NPTXR	O95502	HPA001079	0.003	0.008	0.0005^b^	↓
*Transmembrane protein 132D*	TMEM132D	Q14C87	HPA010739	0.003	0.008	0.0007	↓
*Prodynorphin*	PDYN	P01213	HPA053342	0.003	0.006	0.0007^b^	↓
*Neurocan core protein*	NCAN	O14594	HPA058000	0.003	0.006	0.0007^b^	↓
*Calsyntenin-1*	CLSTN1	O94985	HPA012749^a^	0.003	0.01	0.0009^b^	↓
*Cadherin-8*	CDH8	P55286	HPA014908	0.003	0.01	0.001	↓
*Neural cell adhesion molecule L1-like protein*	CHL1	O00533	HPA003345	0.003	0.02	0.003^b^	↓
*Rabphilin-3A*	RPH3A	Q9Y2J0	HPA002475	0.004	0.01	0.001	↓
*Peptidyl-glycine alpha-amidating monooxygenase*	PAM	P19021	HPA042260^a^	0.004	0.02	0.005^b^	↓
*Neuronal pentraxin-1*	NP1	Q15818	HPA077062	0.006	0.02	0.0007	↓
*von Willebrand factor C domain-containing protein 2-like*	VWC2L	B2RUY7	HPA059414	0.006	0.01	0.003	↑
*Tripeptidyl-peptidase 1*	TPP-1	O14773	HPA037709	0.008	0.02	0.002	↑
*Amyloid-like protein 1*	APLP-1	P51693	HPA028971	0.008	0.05	0.009	↓
*Apolipoprotein A-I*	ApoA-I	P02647	HPA046715^a^	0.01	0.04	0.001	↑
*Neurofilament medium polypeptide*	NF-M	P07197	HPA022845	0.02	0.02	0.0007^b^	↑
*Neurofilament medium polypeptide*	NF-M	P07197	HPA023138	0.02	0.02	0.0008^b^	↑
*Leucine-rich alpha-2-glycoprotein*	LRG	P02750	HPA001888^a^	0.02	0.03	0.002	↑
*Alpha-1-antichymotrypsin*	ACT	P01011	HPA000893^a^	0.02	0.03	0.003	↑
*UPF0606 protein KIAA1549L*	KIAA1549L	Q6ZVL6	HPA051594	0.02	0.02	0.005	↓
*Inter-alpha-trypsin inhibitor heavy chain H1*	ITI-HC1	P19827	HPA042049^a^	0.02	0.03	0.005	↑
*Oligodendrocyte-myelin glycoprotein*	OMG	P23515	HPA008206^a^	0.02	0.02	0.006^b^	↓
*Neuronal cell adhesion molecule*	Nr-CAM	Q92823	HPA061433	0.02	0.02	0.006	↓
*TAR DNA-binding protein 43*	TDP-43	Q13148	HPA070770	0.02	0.01	0.006	↑
*Brevican core protein*	BEHAB	Q96GW7	HPA007865	0.06	0.02	0.008	↓
*Phosphoinositide-3-kinase-interacting protein 1*	PIK3IP1	Q96FE7	HPA007353^a^	0.08	0.04	0.009	↓

P-values (FDR adjusted) based on Mann Whitney U tests for each statistically significant sub group comparison. Group comparisons with PPA (PPA vs NC, PPA vs PMC and PPA vs bvFTD) and between unaffected (NC vs PMC) were not statistically significant

^a^ The generalized linear model showed a significant (*p* < 0.05) age effect

^b^ The result could be replicated in the second cohort

^c^ Direction of change in FTD compared to unaffected individuals

The protein levels were further compared between bvFTD, PPA, PMC and NC. Statistically significant differences (here defined as FDR adjusted *p* < 0.001) in protein levels were found for six proteins between bvFTD and PMC and for fifteen proteins between bvFTD and NC (Table 2). No significant differences were observed when comparing NC with PMC, or when comparing PPA to bvFTD. The most statistically significant differences between the subgroups (bvFTD vs NC and bvFTD vs PMC) were found for the proteins VGF and TN-R. VGF was found in lower levels in bvFTD compared to NC and PMC while TN-R showed the opposite trend (Fig. 3a and f). NF-M showed results similar to that of TNR, with higher levels in FTD patients compared to NC and PMC (Fig. 3e). The proteins NPTXR, TMEM132D and PDYN all displayed lower levels in FTD patients compared to NC and PMC (Fig. 3b-d). A visualisation of the comparisons across the different subgroups for every additional protein in Table 2 can be found in Supplementary Figure 1. Generalized linear models showed that age had a significant effect (*p* < 0.05) on group differences for seven proteins, the same as for the analysis between FTD and unaffected individuals except CLSTN1 (see above).

#### Combining protein levels of VGF, TN-R and NF-M

**Fig. 4:**
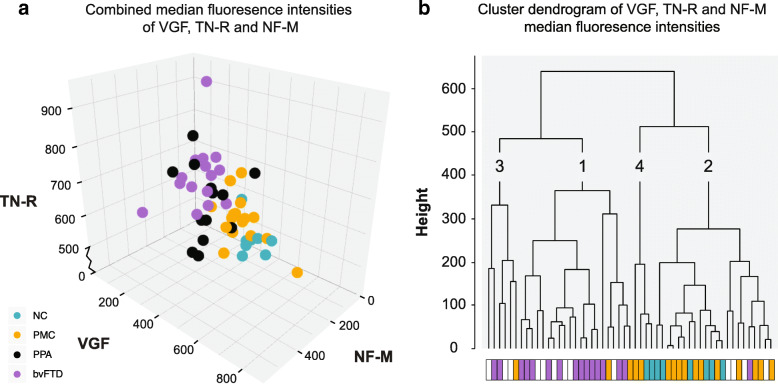
Patient group separations and clustering by VGF, TN-R and NF-M. **a** Separation of FTD patients and unaffected individuals by combining levels of VGF, TN-R and NF-M. NC (turquoise), PMC (yellow), PPA (black for contrast) and bvFTD (purple). **b** Hierarchical clustering of VGF, TN-R and NF-M levels similarly show that FTD patients and unaffected individuals primarily clustered separately. The four groups are indicated by the colored bar, NC (turquoise), PMC (yellow), PPA (white) and bvFTD (purple) and numbers have been added to each cluster for clarification. NC – Non-carriers, PMC – Presymptomatic mutation carriers, PPA – Primary progressive aphasia, bvFTD – Behavioural variant FTD

As previously stated, VGF and TN-R were the two proteins for which the subgroup analyses showed the lowest *p*-values (bvFTD vs NC p-values 0.002 and 0.003 respectively and bvFTD vs PMC p-values 0.0008 and 0.002 respectively) (Table 2). However, in the comparison between all patients with FTD and unaffected individuals, NF-M was one of the proteins with lowest p-value (0.0007) together with a high log2 fold change (0.58). Moreover, NF-M was the only protein with a p-value < 0.001 and log2 fold change > 0.5 that displayed higher levels in FTD compared to unaffected individuals (Fig. 2, Supplementary Table 1). The results on NF-M are highly interesting as they are in agreement with previously published results on the closely related protein neurofilament light chain [19]. Hence, the intensity levels of NF-M, VGF and TN-R were analysed for their combined ability to separate bvFTD, PPA, PMC and NC (Fig. 4). FTD patients could be separated from NC and PMC, as seen in Fig. 4a. Similarly, hierarchical clustering of VGF, TN-R and NF-M protein levels showed that FTD patients and unaffected individuals primarily clustered separately (Fig. 4b). The majority (81%) of the bvFTD patients clustered together (Cluster 1) while most of the PMC (81%) and NC (100%) individuals clustered separately from the patients (Cluster 2 and 4). Patients with PPA were spread out across three clusters, similar to the result from the PCA.

#### Characterization in an independent cohort

**Fig. 5:**
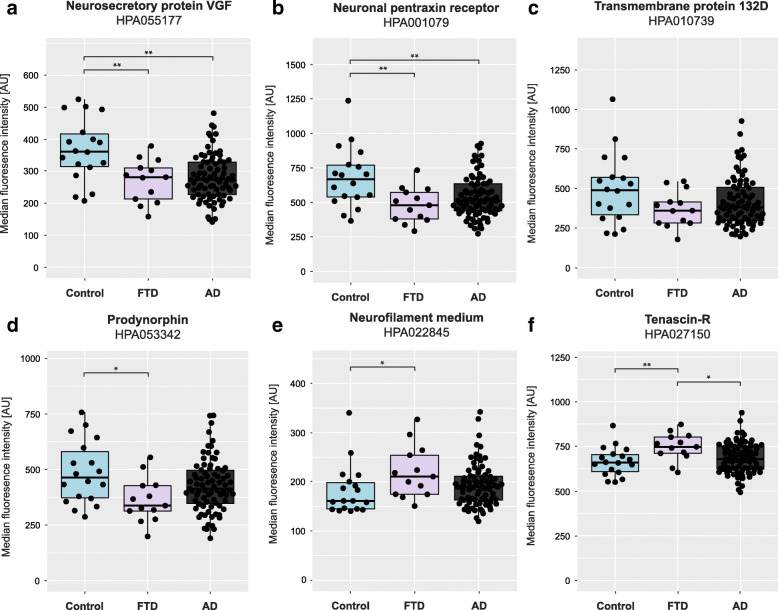
Characterization of VGF, NPTXR, TMEM132D,PDYN, NF-M and TN-R CSF levels in the second cohort. Significant differences between FTD patients and healthy individuals were replicated for (**a**) neurosecretory protein VGF (VGF), (**b**) neuronal pentraxin receptor (NPTXR), (**c**) transmembrane protein 132D (TMEM132D), (**d**) prodynorphin (PDYN), (**e**) neurofilament medium polypeptide (NF-M) and (**f**) tenascin-R (TN-R). Additional differences between FTD and AD was found for TN-R and NF-M (HPA023138, not shown in the figure). Stars indicate significant differences, * *p* < 0.05 and **p < 0.01. Control – healthy individuals, FTD – patients with frontotemporal dementia, AD – patients with Alzheimer’s disease

To investigate whether the differences in protein levels between FTD patients and unaffected individuals (Table 2) could be replicated in another sample material, protein levels were analysed in a second cohort (Table 1). This cohort also included samples from patients with AD to allow comparison to another type of neurodegenerative disease. Results could be replicated in the second cohort for VGF, NPTXR, PDYN, NF-M, TN-R and five other proteins, but not for TMEM132D (Fig. 5, Table 2 and Supplementary Table 3). In addition, a significant difference in protein levels was found between FTD and AD for TN-R and NF-M (only one antibody, HPA023138, Supplementary Table 3), but not for VGF, NPTXR or PDYN. The FTD patients displayed higher levels of both TN-R and NF-M compared to AD. The generalized linear models showed that age had a significant effect (*p* < 0.05) on group differences for three proteins in the second cohort, ACT, LRG and TAR DNA-binding protein 43 (Supplementary Table 2 and Supplementary Table 3).

#### Validation of NF-M antibodies

Since the neurofilament chains contain common domains and sequences, we wanted to confirm that the anti NF-M antibodies did selectively bind NF-M. This was examined by development of sandwich assays using several combinations of antibodies targeting NF-M, NF-L and NF-H (Supplementary Table 4). Results showed that antibody HPA022845 could successfully detect NF-M in combination with the two independent antibodies 20,664–1-AP and 13–0700 (Supplementary Figure 2A-B, for correlation between the sandwich assays see Supplementary Figure 3). In addition, HPA023138 also detected NF-M in combination with a third antibody: 34–1000 (Supplementary Figure 2C). Comparison to single-binder data showed strong correlation (rho> 0.82, *p* < 1E^− 6^) between the two assay formats (Supplementary Figure 2D-F). Correlations between different NF-M assays are found in Supplementary Figure 3*.*

## Discussion

In this study, we found altered protein levels in CSF from FTD patients compared to unaffected individuals. Both principal component analysis and analysis of single protein levels show patterns of separation between unaffected individuals and FTD patients, especially for those with a clinical diagnosis of bvFTD. In total, the levels of 26 proteins in FTD patients were found to be significantly different (*p* < 0.01) when compared to unaffected individuals. When comparing the subgroups NC, PMC, PPA and bvFTD, the most statistically significant differences in protein levels were found for VGF, TN-R, NPTXR, TMEM132D, PDYN and NF-M, none of which were affected by age.

Principal component analysis and hierarchical clustering was used to visualize overall differences on CSF protein levels between the studied individuals. The distribution pattern revealed that the unaffected individuals and FTD patients cluster differently, although with an overlap between the groups. Of the 26 proteins with significantly different levels in FTD patients compared to unaffected individuals, eight displayed an absolute log2 fold change larger than 0.5, also highlighting the overlap between the groups. However, when dividing patients into bvFTD and PPA, and unaffected individuals into PMC and NC, we observed more distinct differences.

A total of 15 proteins showed significant differences between bvFTD and NC and six proteins were significantly different when comparing bvFTD with PMC (Table 2). The direction of each difference was the same when comparing to both NC and PMC, but the PMC protein levels were in general closer to those of bvFTD patients. This trend might illustrate an altered protein profile in the preclinical stage of FTD, similar to what is seen in presymptomatic mutation carriers in genetic AD [31]. The nine proteins that showed a difference between bvFTD and NC, but not between bvFTD and PMC might be important for monitoring the transition to an early disease stage which can be useful for estimation of time to clinical onset. However, the sample size is low, and the PMCs are of different ages, thus with a variable expected time to symptom onset. In the univariate analyses, all PMC individuals are considered as one group when compared to both NC and FTD cases. The differences in time to expected onset within the PMC group may explain why the differences between PMC and NC, as well as bvFTD and PMC for the nine proteins, did not reach statistical significance. On the other hand, the observation that PMC and NC clustered together in the multivariate analysis could imply that a group of proteins, such as the one studied here, will be useful for monitoring the conversion from PMC to FTD. The separation seen between bvFTD and PMC indicates that the protein profile changes at conversion to dementia. This agrees with previous studies on for example NF-L, which is suggested to be a diagnostic, as well as a disease staging marker for FTD [19].

No distinct differences between patients with bvFTD and PPA could be seen neither in the PCA nor the univariate analysis. The large range in protein levels among PPA patients is most likely a contributing factor to the absence of significant differences between PPA and NC/PMC. If this variability is due to the small sample size or in fact reflects a protein heterogeneity in the aphasia phenotype, as could be interpreted by the PCA, has to be further explored.

The most significant differences between bvFTD, NC and PMC were seen for VGF and TN-R. To further examine the protein profiles, all proteins in Table 2 were analysed in a second cohort. The second cohort consisted of both patients with FTD and AD to enable comparisons between different types of neurodegenerative disease. The results from the initial screening could be replicated for ten proteins, although with modest differences. We also found significant differences in TN-R and NF-M levels between FTD and AD patients, suggesting that TN-R and NF-M could be used not only as general markers for neurodegeneration, but more specifically have the potential to separate between different pathologies. However, the differences observed in our study are relatively small and would need to be explored further before their potential can be determined.

VGF is a neuropeptide precursor believed to be important for dendritic growth and neuronal survival [32, 33]. Lower CSF levels of VGF in FTD patients, as seen in our study, was recently demonstrated using mass spectrometry [34–36]. Van der Ende et al. (2019) identified several proteins in CSF that differed between genetic FTD and controls of which seven were selected for validation by parallel reaction monitoring. VGF was found in lower levels in FTD due to *GRN* mutations, compared to controls. Although reaching statistical significance, VGF alone is not enough to distinguish FTD from other dementias as both our own and former studies have shown decreased levels in CSF from AD patients as well [36–39]. TN-R is an extracellular matrix protein expressed by oligodendrocytes and neurons [40, 41]. It is thought to have multiple functions within the central nervous system such as regulation of synaptic plasticity, cell migration and adhesion [42, 43]. Downregulation of hippocampal TN-R was observed in a small set of individuals with AD, compared to age-matched controls by Manavlan et al. (2013) [44] but this is to our knowledge the first study investigating TN-R as a potential biomarker for FTD. TN-R deficient mice display abnormal behaviours and motor coordination [45, 46] which indicates that TN-R is important for maintaining the normal cognitive functions.

In our study, NF-M was found at increased levels in patients which is similar to what has previously been observed for NF-L, with higher levels in FTD patients compared to unaffected individuals [19]. NF-M is approximately twice the size of its well-studied sibling, and assembles into structural filaments together with NF-L and neurofilament heavy chain (NF-H) [47]. The intracellular ratio of the neurofilament isoforms is known [48] but the relative ratios and correlations between isoforms in CSF has not been extensively studied. Although CSF and serum NF-L is known to increase in several neurodegenerative diseases [49, 50] including FTD, NF-M has to our knowledge not been investigated in the context of FTD before. However, high CSF levels of NF-M has previously been reported in stroke patients [51] and using the same NF-M antibodies as in this study, high levels of NF-M were observed in plasma of ALS patients [52].

Promising patterns were also observed for other proteins, such as NPTXR, TMEM132D and PDYN. NPTXR is a receptor protein predominantly expressed in the brain [53]. It is part of the neuronal pentraxin family and has been suggested as a marker for both AD and genetic FTD [34, 39]. NPTXR was recently measured by Van der Ende et al. (2019), together with VGF, and found at lower levels in FTD patients compared to non-carriers, independent of mutation group (*C9orf72*, *GRN* or *MAPT*) [34]. Although different mutation groups could not be compared in our study, we also observed lower levels of NPTXR in patients. TMEM132D is a transmembrane protein also highly expressed in the brain [54]. Its function is still largely unknown but it has been suggested to serve as a cell surface marker for oligodendrocytes [55], and more recently, to possess cell-adhesion functions [56]. Although genetic variants in *TMEM132D* have been associated with primary psychiatric disorders [57], this is to our knowledge, the first report of TMEM132D in relation to FTD, but TMEM132D was one of the proteins for which the results from the first cohort could not be replicated with significance. However, as both cohorts show concordant trends, we still believe TMEM132D to be an interesting protein to be further explored in the context of FTD. PDYN is the precursor of dynorphins, a group of endogenous opioid peptides that has implications for pain and addiction [58]. Certain gene variants of *PDYN* have been associated to episodic memory performance [59] and others have been shown to cause spinocerebellar ataxia type 23 [60]. Yakovleva et al. (2007) reported upregulated levels of dynorphin A (a cleavage product of PDYN), in AD patients but no change in levels of PDYN was observed [61]. Knock-out of PDYN expression was suggested to protect against age related cognitive decline in mice, but its importance in human dementia is still unknown [62].

As this is a pilot study, we acknowledge several limitations. The sample size is small which limits the statistical computations. For example, comparisons between different genetic groups were not possible nor were correlations to time to expected onset in the PMC group (*n* = 16). The presence of motor neuron symptoms could not be assessed separately as they were found in only four cases. Also, only four of the 29 FTD cases were mutation carriers, compared to all of the PMC which might influence the comparisons between these two groups. Since age is the most significant risk factor for dementia, age-related changes in CSF protein concentration is a highly relevant topic for studies of dementia [63–65]. We observed significant age effects on eight of the 26 proteins found to be different in FTD patients compared to unaffected individuals, in any of the two cohorts. Hence, for these eight proteins, we cannot rule out that the differences in protein levels observed between the groups could be due to age rather than diagnosis, or a combination of both. Five of the eight proteins were also among those for which the differences between unaffected individuals and FTD could not be replicated in the second cohort. There could be many reasons why the results could not be replicated, such as sample size and heterogeneity between the two control groups in the patient population, or that there are no generalisable differences between FTD and unaffected individuals for these proteins. Since the control group in the second cohort did not contain any presymptomatic mutation carriers, nothing can be said about the subgroup comparisons. Limitations commonly associated with antibodies such as off-target binding, also applies in this study, and all antibodies have undergone technical validation within the Human Protein Atlas project. The NF-M antibodies have been extensively investigated and the developed sandwich assay shows that both antibodies (HPA023138 and HPA022845) bind NF-M, as previously verified by PRM-mass spectrometry in CSF [66]. Nevertheless, with the setup used here we cannot fully exclude that some reactivity towards either NF-L or NF-H could be present in addition to binding of NF-M. The semi-quantitative nature of the SBA assay could be viewed as a limitation since no definite concentrations are obtained. However, it has previously been shown that the protein levels acquired by the use of suspension bead arrays can be reproduced using other methods [28, 52, 66, 67].

## Conclusions

Overall, our results show that CSF protein profiles differ between bvFTD, PPA, PMC and NC, and that the largest differences are found between bvFTD and NC. Biomarker research in FTD has previously focused primarily on analysis of single proteins and there are limited results on combinations of protein levels, for example NF-L and tau [19]. Here, we demonstrate that it might be possible to separate healthy individuals from patients with FTD by combining the protein levels of VGF, TN-R and NF-M. Promising patterns were also found for a number of other proteins, such as NPTXR, TMEM132D and PDYN, which need to be explored further. We are currently in the process of validating the results in a larger cohort from the GENetic Frontotemporal Dementia Initiative (GENFI) [23], where we include both CSF and plasma samples.

Both clinical phenotype, neuropathology and genetic findings are highly heterogeneous in FTD. With advancements in computational data analysis, we have the possibility to study complex diseases by searching for patterns, as opposed to limiting the analysis to single biomarkers. The results from the PCA and cluster analyses advocate the future use of multivariate methods in dementia research which we plan to incorporate in the follow-up study. Although validation in a larger cohort is necessary to confirm our findings and approach biomarker applications in a clinical setting, we believe the results presented here could be a first step towards highly needed new biomarkers for FTD.

## Supplementary information

**Additional file 1: Supplementary Table 1.** List of 70 proteins included in statistical analysis. Sorted by *p*-value (lowest to highest). **Supplementary Table 2.***P*-values of age effect from generalized linear models. Sorted by order of Table 2. **Supplementary Table 3.** Table of unadjusted *p*-values for group separations in the second cohort. Sorted by order of Table 2. **Supplementary Table 4.** Antibodies used for development of NF-M sandwich assay.

**Additional file 2: Supplementary Figure 1.** CSF levels of proteins in Table 2. Statistically significant differences (p-values) are found in Table 2. NC – Non-carriers, PMC – Presymptomatic mutation carriers, PPA – Primary progressive aphasia, bvFTD – Behavioural variant FTD.

**Additional file 3: Supplementary Figure 2.** Validation of NF-M antibody binding. **(A)** Detection of NF-M with HPA022845 as capture antibody and 20,664–1-AP as detection antibody. No cross-reactivity with antibodies targeting NF-L or NF-H was observed. **(B)** Detection of NF-M with HPA022845 as capture antibody and 13–0700 as detection antibody. No cross-reactivity with antibodies targeting NF-L or NF-H was observed. **(C)** Detection of NF-M with HPA023138 as capture antibody and 34–1000 as detection antibody. No cross-reactivity with antibodies targeting NF-L or NF-H was observed. **(D-F)** Comparison between sandwich assay data and single-binder data. Rho_(D)_ = 0.85, p_(D)_ = 1E^− 7^, rho_(E)_ = 0.86, p_(E)_ = 1E^− 7^, rho_(F)_ = 0.82, p_(F)_ = 1E^− 6^.

**Additional file 4: Supplementary Figure 3.** Correlation between NF-M assays. **(A)** Correlation between HPA022845 and HPA023138 single-binder data. **(B)** Correlation between HPA022845 sandwich assays. HPA022845 was used as capture antibody together with two different detection antibodies, 20,664–1-AP and 13–0700. **(C)** Correlation between one HPA22845 sandwich assay and HPA023138 sandwich assay. HPA022845 was used as capture antibody together with 20,664–1-AP as detection antibody, and HPA023138 was used as capture antibody together with 34–1000 as detection antibody.

**Additional file 5: Supplementary Figure 4.** Antibodies used for development of NF-M sandwich assay aligned to the neurofilament amino acid sequences. The neurofilaments are shown in green and the size of the domains (head, rod and tail) are displayed at the top. The highly conserved rod domain is highlighted in grey. Position and length of the amino acid sequences used to generate the antibodies are shown in blue and the exact amino acid positions are given in brackets, if known. Antibody 13–1300 targets all three neurofilaments. The smallest epitope identified for HPA022845 corresponds to amino acids 746–749 [50].

## Data Availability

The datasets generated and analysed during the current study are available from the corresponding authors upon request.

## Abbreviations

ACT: Alpha-1-antichymotrypsin
AD: Alzheimer’s disease
ALS: Amyotrophic lateral sclerosis
ApoA-I: Apolipoprotein A-I
bvFTD: Behavioural variant FTD
CDH8: Cadherin-8
CHL1: Neural cell adhesion molecule L1-like protein
CLSTN1: Calsyntenin-1
CSF: Cerebrospinal fluid
CV: Coefficient of variance
FDR: False discovery rate
FTD: Frontotemporal dementia
GENFI: GENetic frontotemporal dementia initiative
GRN: Progranulin
HPA: Human protein atlas project
LCCC: Lin’s concordance coefficient
LRG: Leucine-rich alpha-2-glycoprotein
MAPT: Microtubule-associated protein tau
MFI: Median fluorescence intensity
MMSE: Mini-mental State examination
NBB: Netherlands institute for neuroscience
NC: Non-carriers
NCAN: Neurocan core protein
NF-H: Neurofilament heavy chain
NF-L: Neurofilament light chain
NF-M: Neurofilament medium polypeptide
NINCDS-ADRDA: National institute of neurological and communicative disorders and stroke and the Alzheimer’s disease and related disorders association
NP1: Neuronal pentraxin-1
NPTXR: Neuronal pentraxin receptor
PBS-T: Phosphate buffered saline with 0.05% Tween® 20
PCA: Principal component analysis
PDYN: Prodynorphin
PMC: Presymptomatic mutation carriers
PNFA: Progressive non-fluent aphasia
PPA: Progressive primary aphasia
SAPE: Streptavidin coupled fluorophore
SD: Semantic dementia
TMEM132D: Transmembrane protein 132D
TN-R: Tenascin-R
VCP: Valosin containing protein
VGF: Neurosecretory protein VGF
