# Supplementary material for: Altered levels of CSF proteins in patients with FTD, presymptomatic mutation carriers and non-carriers
Source: Transl Neurodegener. 2020 Jun 23;9:27. doi: 10.1186/s40035-020-00198-y (PMC7310563; doi:10.1186/s40035-020-00198-y)
Supplement: Supplementary file 1 — Additional file 1: Supplementary Table 1. List of 70 proteins included in statistical analysis. Sorted by p-value (lowest to highest). Supplementary Table 2.P-values of age effect from generalized linear models. Sorted by order of Table 2. Supplementary Table 3. Table of unadjusted p-values for group separations in the second cohort. Sorted by order of Table 2. Supplementary Table 4. Antibodies used for development of NF-M sandwich assay. [file 40035_2020_198_MOESM1_ESM.docx]

# Supplementary Materials

## Participants

An independent cohort from Uppsala University Hospital was used to replicate the main findings in the cohort collected at the Karolinska University Hospital Memory clinic. Patients were recruited at the Uppsala University Hospital and control subjects were recruited through advertisements in the local newspaper. FTD was diagnosed in patients with frontal symptoms (disinhibition, apathy, loss of empathy, perseverations or hyperorality), with evidence of frontotemporal atrophy or reduced glucose uptake in these regions as measured with positron emission tomography, and according to the McKhann criteria or Rascovsky criteria(1,2). An AD diagnosis was established according to NINCDS-ADRDA (National Institute of Neurological and Communicative Disorders and Stroke and the Alzheimer’s Disease and Related Disorders Association) criteria(3) and the Diagnostic and Statistical Manual of Mental Disorders, Fourth Edition, criteria(4). Control subjects were determined cognitively unimpaired, based on their history, a Mini-Mental State Examination (MMSE) test and no abnormal radiological findings in computerized tomography of the brain.

## Sample collection

At the Karolinska University Hospital Memory clinic, CSF samples were collected between 1997 and 2016 by lumbar puncture into polypropylene tubes. Immediately after collection, the fluid was centrifuged at 963 xg for 10 minutes at 4°C. The supernatant was aliquoted into 2 ml polypropylene cryotubes and stored at -80°C. Peripheral blood was collected by venipuncture. Genomic DNA was isolated with Gentra Puregene Blood kit (Qiagen) according to manufacturers’ protocol and stored at -80 C.

At the Uppsala University Hospital, CSF samples were collected by lumbar puncture between 2008 and 2014 into polypropylene tubes. Immediately after collection, the fluid was centrifuged at 1300 xg for 10 minutes at 4°C. All samples were visually inspected for blood contamination, frozen and stored at -80°C.

## Genetic screening

FTD patients recruited at the Karolinska University Hospital Memory clinic were screened for mutations in *MAPT* and *GRN* by Sanger-sequencing as described in Öijerstedt et al. 2019 (n=25) or by whole genome sequencing (n=4) and confirmation by Sanger sequencing. For whole genome sequencing, library preparation was done using the TruSeq DNA PCR-free kit (Illumina) and sequencing was done on HiSeq (2x150bp reads, 1 lane per library, Illumina). The *C9orf72* repeat expansion was identified by repeat primed PCR and short tandem repeat(5,6). At-risk participants were screened for the mutation segregating in the family using Sanger-sequencing (for *MAPT* and *GRN*) or repeat primed PCR (for *C9orf72* repeat expansion).

## Protein profiling

Antibody suspension bead arrays were used to explore the protein profiles in human CSF. The antibodies used for profiling were selected based upon previously published and unpublished data generated within projects on various types of dementia and neurodegenerative disorders(7). Among these, were antibodies targeting proteins known to be associated to Alzheimer’s disease (AD), Parkinson’s disease, Lewy body dementia and ALS, and proteins that were selected on the basis of RNA levels measured in brain(8) or due to a known, suggested or probable relevance to FTD. A total of 328 proteins, targeted by 492 antibodies, were included in the experimental analysis. The antibodies were polyclonal rabbit IgG antibodies created within the Human Protein Atlas (HPA, www.proteinatlas.org).

Three assay specific control (Rabbit IgG, anti-human IgG and anti-human albumin) antibodies were included for evaluation of background binding and sample load. Rabbit IgG (P120-301, Bethyl laboratories) was used to measure inherent binding to rabbit IgG molecules. Anti-human IgG (309-005-082, Jackson) and anti-human albumin (A0001, Dako) were used to measure sample load. Blocked bare beads were used for measuring background binding to the bead surface.

Experimental procedure. Creation of the suspension bead array was done by immobilizing 1.75 µg of antibodies onto magnetic, color coded carboxylated beads (MagPlex-C, Luminex Corp) as described elsewhere(9,10). Fifteen µl of each CSF sample was aliquoted to 96-well plates in a structured distribution. To enable detection by a streptavidin coupled fluorophore (SAPE) (SA1004-4, Invitrogen), the samples were diluted 1/2 and labeled with a ﻿tenfold molar excess (0.5 mg/ml) of biotin (21329, Thermo Scientific) as described previously(10,11). The labelled samples were further diluted 1/8 in assay buffer (1× PBS with 0.5% (w/v) polyvinyl alcohol (P8136, Sigma-Aldrich), 0.8% (w/v) polyvinylpyrrolidone (PVP360, Sigma Life Science), 0.1% casein (C5890, Sigma Life Science) and 0.5 mg/ml rabbit IgG (P120-301, Bethyl Laboratories)) and heat treated at 56°C for 30 min in a water bath (TW8, Julabo). After 10 min cool down on the bench, 45 µl of each sample was transferred to 5 µl of suspension bead array using a liquid handler (SELMA, CyBio) and incubated overnight at ambient temperature. Following incubation, the beads were washed with 3x100 µl of phosphate buffered saline with 0.05% Tween® 20 (PBS-T), incubated with 50 µl 0.4% paraformaldehyde for 10 min, washed again with 3x100 µl of PBS-T, incubated with 50 µl of 0.5 µg/mL SAPE for 20 min and finally washed with 3x100 µl of PBS-T one last time. The beads were then resuspended in 60 µl before detection of captured proteins in a FlexMap3D instrument (Luminex Corporation). Results were reported as median fluorescence intensities (MFI) per bead identity and sample, calculated from at least 30 measured beads. All incubations were done at room temperature on shaker, if nothing else is stated. To assess inter-assay reproducibility, a subset of the antibody arrays was run twice using re-biotinylated samples.

## Validation of NF-M antibodies

To examine potential off-target binding for the antibodies raised against neurofilaments, a sandwich assay directed toward neurofilament medium polypeptide (NF-M) was developed according to a previously published workflow(12). Six NF-M antibodies were tested in combination, together with five antibodies targeting both NF-L and neurofilament heavy chain (NF-H) (Supplementary Table 3 and Supplementary Figure 4). Capture antibodies were immobilized onto magnetic color-coded beads as mentioned above (section *Protein profiling*). Detection antibodies were biotinylated using bead-assisted labeling(13). One µg antibody diluted in 30 µl PBS-T was added to 5 µl beads (Dynabeads Protein G, Invitrogen, 30 mg/ml) and incubated for 30 min. The beads were then washed with 2x100 µl of PBS-T before incubation with labeling reagent (2.2 µl biotin (0.5 mg/ml in DMSO) + 60 µl PBS-T) for 30 min. Following a wash with 3x100 µl of PBS-T, the labeled antibodies were eluted for 2 min with elution buffer (Acetate 2.0 M pH 3.2) and 2 µl of Tris (1 M, pH 8) was added to stop the labeling reaction. Finally, 3 µl of PBS-T was added to the labeled antibodies before storage at 4°C.

In order to validate the antibody binding 24 CSF samples collected post mortem, from the Netherlands Brain Bank, Netherlands Institute for Neuroscience (NBB) were analyzed. The sample set included healthy individuals (n=8), patients with Alzheimer’s disease (n=10) and Lewy body dementia (n=6) and was collected according to the Ethical and legal declaration of the NBB (www.brainbank.nl).

Experimental procedure. CSF samples were diluted 1/2 by mixing 25 µl of CSF with 25 µl assay buffer. The samples were then heat treated as mentioned previously (section *Protein profiling*) and 45 µl of each sample was transferred to 5 µl of 3x concentrated suspension bead array (to decrease the sample volume needed) before incubation overnight. Following incubation, the beads were washed with 3x100 µl of PBS-T. To allow testing of three detection antibodies in one experiment the beads in each well were divided into three. This was done by resuspending the beads in 100 µl of PBS-T and transferring 2x33 µl suspended beads to two new wells. The detection antibodies were then diluted to a concentration of 1 µg/ml in PBS-T and 25 µl was added to each well. After 2 hours incubation the beads were washed again with 3x100 µl of PBS-T, incubated with 50 µl of 0.5 µg/mL SAPE for 20 min and finally washed with 3x100 µl of PBS-T one last time. The beads were then resuspended in 100 µl before detection of captured proteins in the FlexMap3D instrument. All incubations were done at room temperature on shaker, if nothing else is stated.

## Quality control and reproducibility

Four assay specific control antibodies were included to evaluate background binding and sample load. The mean background binding in each assay was determined by calculating the mean of the empty beads included in the analysis. The mean background for each sample was then subtracted from all other MFI signals of that sample before further investigation. All antibodies with MFI signals below the Rabbit IgG bead were removed from analysis. Signals from the loading controls (anti-human IgG and anti-human albumin) were used to identify wells for potential exclusion from analysis. No wells were excluded from the analysis.

To assess the intra-assay reproducibility, three identical wells of a sample pool were included in the assay plate and coefficients of variation (CV) were calculated for each bead ID. The median CV within each assay was between 4.6-5.8% (IQR=3.3-4.6). Lin’s concordance coefficient (LCCC) (14) was calculated for the re-run assay and showed a substantial concordance of 0.976 (95% CI=0.976-0.977)(15). To further investigate the inter-assay reproducibility, the overall spearman correlation between the assays was calculated to 0.94. For assessment of inter-assay reproducibility, Lin’s concordance correlation coefficient (LCCC) was calculated using ‘CCC’ {DescTools}. All correlation coefficients for inter-assay reproducibility were calculated using Spearman’s rho statistics.

# Supplementary Tables

## Supplementary Table 1. List of 70 proteins included in statistical analysis. Sorted by p-value (lowest to highest).

| **Protein name** | **Short name** | **Uniprot ID** | **Antibody** | **p** | **log2 (fold change)** | **Direction of change^a^** |
| --- | --- | --- | --- | --- | --- | --- |
| *Neurosecretory protein VGF* | VGF | O15240 | HPA055177 | 0,0001 | -0,67 | ↓ |
| *Neuronal pentraxin receptor* | NPTXR | O95502 | HPA001079 | 0,0005 | -0,76 | ↓ |
| *Transmembrane protein 132D* | TMEM132D | Q14C87 | HPA010739 | 0,0007 | -0,83 | ↓ |
| *Prodynorphin* | PDYN | P01213 | HPA053342 | 0,0007 | -0,62 | ↓ |
| *Neurofilament medium polypeptide* | NF-M | P07197 | HPA022845 | 0,0007 | 0,58 | ↑ |
| *Tenascin-R* | TN-R | Q92752 | HPA027150 | 0,0007 | 0,32 | ↑ |
| *Neuronal pentraxin-1* | NP1 | Q15818 | HPA077062 | 0,0007 | -0,29 | ↓ |
| *Neurocan core protein* | NCAN | O14594 | HPA058000 | 0,0007 | -0,26 | ↓ |
| *Neurofilament medium polypeptide* | NF-M | P07197 | HPA023138 | 0,0008 | 0,64 | ↑ |
| *Calsyntenin-1* | CLSTN1 | O94985 | HPA012749 | 0,0009 | -0,29 | ↓ |
| *Apolipoprotein A-I* | ApoA-I | P02647 | HPA046715 | 0,001 | 0,61 | ↑ |
| *Cadherin-8* | CDH8 | P55286 | HPA014908 | 0,001 | -0,52 | ↓ |
| *Rabphilin-3A* | RPH3A | Q9Y2J0 | HPA002475 | 0,001 | -0,47 | ↓ |
| *Leucine-rich alpha-2-glycoprotein* | LRG | P02750 | HPA001888 | 0,002 | 0,25 | ↑ |
| *Tripeptidyl-peptidase 1* | TPP-1 | O14773 | HPA037709 | 0,002 | 0,24 | ↑ |
| *Neural cell adhesion molecule L1-like protein* | CHL1 | O00533 | HPA003345 | 0,003 | -0,51 | ↓ |
| *Alpha-1-antichymotrypsin* | ACT | P01011 | HPA000893 | 0,003 | 0,33 | ↑ |
| *von Willebrand factor C domain-containing protein 2-like* | VWC2L | B2RUY7 | HPA059414 | 0,003 | 0,16 | ↑ |
| *Peptidyl-glycine alpha-amidating monooxygenase* | PAM | P19021 | HPA042260 | 0,005 | -0,27 | ↓ |
| *UPF0606 protein KIAA1549L* | KIAA1549L | Q6ZVL6 | HPA051594 | 0,005 | -0,22 | ↓ |
| *Inter-alpha-trypsin inhibitor heavy chain H1* | ITI-HC1 | P19827 | HPA042049 | 0,005 | 0,16 | ↑ |
| *Neuronal cell adhesion molecule* | Nr-CAM | Q92823 | HPA061433 | 0,006 | -0,38 | ↓ |
| *Oligodendrocyte-myelin glycoprotein* | OMG | P23515 | HPA008206 | 0,006 | -0,36 | ↓ |
| *TAR DNA-binding protein 43* | TDP-43 | Q13148 | HPA070770 | 0,006 | 0,29 | ↑ |
| *Brevican core protein* | BEHAB | Q96GW7 | HPA007865 | 0,008 | -0,18 | ↓ |
| *Phosphoinositide-3-kinase-interacting protein 1* | PIK3IP1 | Q96FE7 | HPA007353 | 0,009 | -0,15 | ↓ |
| *Amyloid-like protein 1* | APLP-1 | P51693 | HPA028971 | 0,009 | -0,08 | ↓ |
| *Voltage-dependent calcium channel subunit alpha-2/delta-1* | CACNA2D1 | P54289 | HPA008213 | 0,01 | -0,31 | ↓ |
| *Regulating synaptic membrane exocytosis protein 3* | Nim3 | Q9UJD0 | HPA055285 | 0,01 | 0,30 | ↑ |
| *Complement component C9* | C9 | P02748 | HPA029577 | 0,01 | 0,29 | ↑ |
| *RING finger protein 175* | RNF175 | Q8N4F7 | HPA028830 | 0,01 | 0,17 | ↑ |
| *Apolipoprotein A-IV* | ApoA-IV | P06727 | HPA001352 | 0,02 | 0,28 | ↑ |
| *Afamin* | AFM | P43652 | HPA052437 | 0,02 | 0,22 | ↑ |
| *Cell adhesion molecule 2* | CADM2 | Q8N3J6 | HPA010024 | 0,02 | -0,20 | ↓ |
| *Brain acid soluble protein 1* | BASP1 | P80723 | HPA050333 | 0,02 | -0,20 | ↓ |
| *Receptor expression-enhancing protein 2* | REEP2 | Q9BRK0 | HPA031813 | 0,03 | -0,16 | ↓ |
| *Neurofascin* | NFASC | O94856 | HPA073444 | 0,04 | -0,24 | ↓ |
| *Secretogranin-3* | SgIII | Q8WXD2 | HPA053715 | 0,04 | -0,22 | ↓ |
| *Fibrinogen alpha chain* | FGA | P02671 | HPA051370 | 0,04 | 0,19 | ↑ |
| *Multiple EGF-like domains protein 10* | MEGF10 | Q96KG7 | HPA026876 | 0,04 | -0,13 | ↓ |
| *Vesicular glutamate transporter 2* | VGluT2 | Q9P2U8 | HPA039226 | 0,04 | 0,11 | ↑ |
| *Lymphocyte antigen 6H* | Ly-6H | O94772 | HPA077218 | 0,06 | -0,27 | ↓ |
| *Neuromodulin* | GAP43 | P17677 | HPA013392 | 0,07 | -0,23 | ↓ |
| *Progranulin* | PGRN | P28799 | HPA028747 | 0,07 | 0,19 | ↑ |
| *Pro-opiomelanocortin* | POMC | P01189 | HPA063644 | 0,08 | -0,23 | ↓ |
| *Phosphatidylethanolamine-binding protein 1* | PEBP-1 | P30086 | HPA063904 | 0,08 | -0,09 | ↓ |
| *Endoplasmin* | GRP-94 | P14625 | HPA003901 | 0,1 | -0,25 | ↓ |
| *Semaphorin-7A* | SEMA7A | O75326 | HPA042273 | 0,1 | -0,18 | ↓ |
| *Beta-synuclein* | SNCB | Q16143 | HPA035876 | 0,1 | -0,18 | ↓ |
| *Dimethylarginine dimethylaminohydrolase 1* | DDAH-1 | O94760 | HPA006308 | 0,1 | -0,16 | ↓ |
| *Interleukin-6 receptor subunit beta* | IL-6RB | P40189 | HPA010030 | 0,1 | -0,15 | ↓ |
| *Cell cycle exit and neuronal differentiation protein 1* | CEND1 | Q8N111 | HPA042527 | 0,1 | -0,11 | ↓ |
| *Insulin-like growth factor-binding protein 6* | IGFBP-6 | P24592 | HPA075088 | 0,1 | 0,09 | ↑ |
| *Ermin* | ERMN | Q8TAM6 | HPA038295 | 0,3 | 0,11 | ↑ |
| *Myelin basic protein* | MBP | P02686 | HPA049222 | 0,3 | 0,10 | ↑ |
| *Serum paraoxonase/arylesterase 1* | PON 1 | P27169 | HPA001640 | 0,3 | 0,10 | ↑ |
| *Myelin-oligodendrocyte glycoprotein* | MOG | Q16653 | HPA021873 | 0,3 | -0,09 | ↓ |
| *Dyslexia-associated protein KIAA0319* | KIAA0319 | Q5VV43 | HPA015607 | 0,3 | -0,06 | ↓ |
| *Synaptotagmin-11* | SytXI | Q9BT88 | HPA064091 | 0,3 | -0,02 | ↓ |
| *Amphiphysin* | AMPH | P49418 | HPA019828 | 0,4 | -0,04 | ↓ |
| *Selenoprotein P* | SeP | P49908 | HPA058160 | 0,5 | 0,00 | ↓ |
| *Vasorin* | VASN | Q6EMK4 | HPA011246 | 0,6 | -0,06 | ↓ |
| *Extracellular matrix protein 1* | ECM1 | Q16610 | HPA027241 | 0,6 | -0,04 | ↓ |
| *Membrane progestin receptor delta* | mPR delta | Q6TCH4 | HPA010843 | 0,7 | -0,17 | ↓ |
| *Dickkopf-related protein 3* | Dkk-3 | Q9UBP4 | HPA011164 | 0,7 | -0,08 | ↓ |
| *Monocyte differentiation antigen CD14* | CD14 | P08571 | HPA002035 | 0,7 | 0,06 | ↑ |
| *Interleukin-1 receptor accessory protein-like 1* | IL1RAPL-1 | Q9NZN1 | HPA000564 | 0,7 | 0,00 | ↓ |
| *Leucine-rich repeat-containing protein 4B* | LRRC4B | Q9NT99 | HPA058986 | 0,9 | -0,32 | ↓ |
| *ICOS ligand* | ICOSLG | O75144 | HPA070452 | 0,9 | 0,18 | ↑ |
| *GABA aminotransferase* | GABA-AT | P80404 | HPA041528 | 0,9 | -0,10 | ↓ |
| *Aquaporin-4* | AQP-4 | P55087 | HPA014784 | 1 | 0,12 | ↑ |

P-values (FDR adjusted) based on Mann Whitney U tests for each statistically significant sub group comparison.

^a^ Direction of change in FTD compared to unaffected individuals.

## Supplementary Table 2. P-values of age effect from generalized linear models. Sorted by order of Table 2.

| **Protein name** | **Short name** | **Antibody** | **Cohort 1**  Two groups | **Cohort 1**  Four groups | **Cohort 2** |
| --- | --- | --- | --- | --- | --- |
| *Neurosecretory protein VGF* | VGF | HPA055177 | 0.2 | 0.3 | 0.6 |
| *Tenascin-R* | TN-R | HPA027150 | 0.9 | 0.7 | 0.07 |
| *Neuronal pentraxin receptor* | NPTXR | HPA001079 | 0.1 | 0.2 | 0.5 |
| *Transmembrane protein 132D* | TMEM132D | HPA010739 | 0.4 | 0.5 | 0.9 |
| *Prodynorphin* | PDYN | HPA053342 | 0.2 | 0.3 | 0.8 |
| *Neurocan core protein* | NCAN | HPA058000 | 0.7 | 0.8 | 0.9 |
| *Calsyntenin-1* | CLSTN1 | HPA012749 | 0.04 | 0.07 | 0.4 |
| *Cadherin-8* | CDH8 | HPA014908 | 0.7 | 0.8 | 0.8 |
| *Neural cell adhesion molecule L1-like protein* | CHL1 | HPA003345 | 0.2 | 0.3 | 0.9 |
| *Rabphilin-3A* | RPH3A | HPA002475 | 0.08 | 0.1 | 0.7 |
| *Peptidyl-glycine alpha-amidating monooxygenase* | PAM | HPA042260 | 0.02 | 0.03 | 0.4 |
| *Neuronal pentraxin-1* | NP1 | HPA077062 | 0.2 | 0.2 | 0.5 |
| *von Willebrand factor C domain-containing protein 2-like* | VWC2L | HPA059414 | 0.2 | 0.07 | 0.12 |
| *Tripeptidyl-peptidase 1* | TPP-1 | HPA037709 | 0.1 | 0.08 | 0.1 |
| *Amyloid-like protein 1* | APLP-1 | HPA028971 | 0.2 | 0.3 | 0.4 |
| *Apolipoprotein A-I* | ApoA-I | HPA046715 | 0.04 | 0.03 | 0.2 |
| *Neurofilament medium polypeptide* | NF-M | HPA022845 | 0.05 | 0.08 | 0.3 |
| *Neurofilament medium polypeptide* | NF-M | HPA023138 | 0.4 | 0.7 | 0.2 |
| *Leucine-rich alpha-2-glycoprotein* | LRG | HPA001888 | 0.00006 | 0.00006 | 0.004 |
| *Alpha-1-antichymotrypsin* | ACT | HPA000893 | 0.00006 | 0.0001 | 0.002 |
| *UPF0606 protein KIAA1549L* | KIAA1549L | HPA051594 | 0.1 | 0.2 | 0.3 |
| *Inter-alpha-trypsin inhibitor heavy chain H1* | ITI-HC1 | HPA042049 | 0.03 | 0.03 | 0.8 |
| *Oligodendrocyte-myelin glycoprotein* | OMG | HPA008206 | 0.01 | 0.02 | 0.4 |
| *Neuronal cell adhesion molecule* | Nr-CAM | HPA061433 | 0.09 | 0.1 | 0.6 |
| *TAR DNA-binding protein 43* | TDP-43 | HPA070770 | 0.2 | 0.08 | 0.006 |
| *Brevican core protein* | BEHAB | HPA007865 | 0.2 | 0.2 | 0.9 |
| *Phosphoinositide-3-kinase-interacting protein 1* | PIK3IP1 | HPA007353 | 0.007 | 0.01 | 0.1 |

## Supplementary Table 3. Table of unadjusted p-values for group separations in the second cohort. Sorted by order of Table 2.

| **Protein name** | **Short name** | **Antibody** | **FTD vs AD** | **FTD vs Controls** | **AD vs Controls** |
| --- | --- | --- | --- | --- | --- |
| *Neurosecretory protein VGF* | VGF | HPA055177 | 0.8 | 0.004 | 0.0006 |
| *Tenascin-R* | TN-R | HPA027150 | 0.02 | 0.006 | 0.3 |
| *Neuronal pentraxin receptor* | NPTXR | HPA001079 | 0.2 | 0.005 | 0.007 |
| *Transmembrane protein 132D* | TMEM132D | HPA010739 | 0.5 | 0.1 | 0.2 |
| *Prodynorphin* | PDYN | HPA053342 | 0.05 | 0.01 | 0.1 |
| *Neurocan core protein* | NCAN | HPA058000 | 0.2 | 0.007 | 0.3 |
| *Calsyntenin-1* | CLSTN1 | HPA012749 | 0.1 | 0.01 | 0.02 |
| *Cadherin-8* | CDH8 | HPA014908 | 0.6 | 0.09 | 0.04 |
| *Neural cell adhesion molecule L1-like protein* | CHL1 | HPA003345 | 0.4 | 0.02 | 0.03 |
| *Rabphilin-3A* | RPH3A | HPA002475 | 0.2 | 0.2 | 0.3 |
| *Peptidyl-glycine alpha-amidating monooxygenase* | PAM | HPA042260 | 0.7 | 0.03 | 0.01 |
| *Neuronal pentraxin-1* | NP1 | HPA077062 | 0.4 | 0.09 | 0.1 |
| *von Willebrand factor C domain-containing protein 2-like* | VWC2L | HPA059414 | 0.2 | 0.1 | 0.7 |
| *Tripeptidyl-peptidase 1* | TPP-1 | HPA037709 | 0.2 | 0.2 | 0.9 |
| *Amyloid-like protein 1* | APLP-1 | HPA028971 | 0.3 | 0.6 | 0.05 |
| *Apolipoprotein A-I* | ApoA-I | HPA046715 | 0.2 | 0.5 | 0.7 |
| *Neurofilament medium polypeptide* | NF-M | HPA022845 | 0.07 | 0.02 | 0.1 |
| *Neurofilament medium polypeptide* | NF-M | HPA023138 | 0.004 | 0.0004 | 0.4 |
| *Leucine-rich alpha-2-glycoprotein* | LRG | HPA001888^a^ | 0.1 | 0.3 | 0.9 |
| *Alpha-1-antichymotrypsin* | ACT | HPA000893^a^ | 0.06 | 0.1 | 0.8 |
| *UPF0606 protein KIAA1549L* | KIAA1549L | HPA051594 | 0.9 | 0.2 | 0.1 |
| *Inter-alpha-trypsin inhibitor heavy chain H1* | ITI-HC1 | HPA042049 | 0.2 | 0.06 | 0.3 |
| *Oligodendrocyte-myelin glycoprotein* | OMG | HPA008206 | 0.2 | 0.01 | 0.01 |
| *Neuronal cell adhesion molecule* | Nr-CAM | HPA061433 | 0.9 | 0.09 | 0.03 |
| *TAR DNA-binding protein 43* | TDP-43 | HPA070770^a^ | 0.1 | 0.4 | 0.4 |
| *Complement component C9* | C9 | HPA070709^a^ | 0.08 | 0.1 | 1 |
| *Brevican core protein* | BEHAB | HPA007865 | 0.7 | 0.1 | 0.04 |
| *Phosphoinositide-3-kinase-interacting protein 1* | PIK3IP1 | HPA007353 | 0.7 | 0.2 | 0.2 |

^a^ The generalized linear model showed a significant (p < 0.05) age effect.

## Supplementary Table 4. Antibodies used for development of NF-M sandwich assay.

| **Antibody name** | **Provider** | **Target protein** | **Species and clonality** |
| --- | --- | --- | --- |
| HPA014850 | Atlas Antibodies | NF-L | Rabbit polyclonal |
| MAB2216 | R&D Systems, Bio-Techne | NF-L | Mouse monoclonal |
| HPA022845 | Atlas Antibodies | NF-M | Rabbit polyclonal |
| HPA023138 | Atlas Antibodies | NF-M | Rabbit polyclonal |
| MAB3029 | R&D Systems, Bio-Techne | NF-M | Mouse monoclonal |
| 20664-1-AP | Nordic Biosite | NF-M | Rabbit polyclonal |
| 13-0700 | Thermo Fisher Scientific | NF-M | Mouse monoclonal |
| 34-1000 | Thermo Fisher Scientific | NF-M | Mouse monoclonal |
| HPA061615 | Atlas Antibodies | NF-H | Rabbit polyclonal |
| MAB3108 | R&D Systems, Bio-Techne | NF-H | Mouse monoclonal |
| 13-1300 | Thermo Fisher Scientific | NF-L/M/H | Mouse monoclonal |

# Supplementary reference list

1. McKhann GM, Albert MS, Grossman M, Miller B, Dickson D, Trojanowski JQ. Clinical and pathological diagnosis of frontotemporal dementia: Report of the work group on Frontotemporal Dementia and Pick’s Disease. Arch Neurol. 2001;

2. Rascovsky K, Hodges JR, Knopman D, Mendez MF, Kramer JH, Neuhaus J, et al. Sensitivity of revised diagnostic criteria for the behavioural variant of frontotemporal dementia. Brain [Internet]. 2011 Sep;134(9):2456–77. Available from: https://academic.oup.com/brain/article-lookup/doi/10.1093/brain/awr179

3. McKhann G, Drachman D, Folstein M, Katzman R, Price D, Stadlan EM. Clinical diagnosis of Alzheimer’s disease: report of the NINCDS-ADRDA Work Group under the auspices of Department of Health and Human Services Task Force on Alzheimer’s Disease. Neurology. 1984 Jul;34(7):939–44.

4. Bell CC. DSM-IV: Diagnostic and Statistical Manual of Mental Disorders. JAMA [Internet]. 1994 Sep 14;272(10):828–9. Available from: https://doi.org/10.1001/jama.1994.03520100096046

5. Gijselinck I, Van Langenhove T, van der Zee J, Sleegers K, Philtjens S, Kleinberger G, et al. A C9orf72 promoter repeat expansion in a Flanders-Belgian cohort with disorders of the frontotemporal lobar degeneration-amyotrophic lateral sclerosis spectrum: A gene identification study. Lancet Neurol. 2012;

6. Öijerstedt L, Chiang H-H, Björkström J, Forsell C, Lilius L, Lindström A-K, et al. Confirmation of high frequency of C9orf72 mutations in patients with frontotemporal dementia from Sweden. Neurobiol Aging [Internet]. 2019 Mar [cited 2019 Sep 4]; Available from: https://linkinghub.elsevier.com/retrieve/pii/S0197458019300879

7. Remnestål J, Just D, Mitsios N, Fredolini C, Mulder J, Schwenk JM, et al. CSF profiling of the human brain enriched proteome reveals associations of neuromodulin and neurogranin to Alzheimer’s disease. Proteomics - Clin Appl. 2016;10(12):1242–53.

8. Sjöstedt E, Fagerberg L, Hallström BM, Ha¨ggmark A, Mitsios N, Nilsson P, et al. Defining the human brain proteome using transcriptomics and antibody-based profiling with a focus on the cerebral cortex. PLoS One. 2015;10(6):1–20.

9. Schwenk JM, Gry M, Rimini R, Uhlén M, Nilsson P. Antibody suspension bead arrays – An application for serum and plasma analysis within antibody proteomics. J Proteome Res. 2008;7:3168–79.

10. Pin E, Sjoberg R, Andersson E, Hellstrom C, Olofsson J, Jernbom Falk A, et al. Array-Based Profiling of Proteins and Autoantibody Repertoires in CSF. Methods Mol Biol. 2019;2044:303–18.

11. Häggmark A, Byström S, Ayoglu B, Qundos U, Uhlén M, Khademi M, et al. Antibody-based profiling of cerebrospinal fluid within multiple sclerosis. Proteomics. 2013;13(15):2256–67.

12. Haussler RS, Bendes A, Iglesias M, Sanchez-Rivera L, Dodig-Crnkovic T, Bystrom S, et al. Systematic Development of Sandwich Immunoassays for the Plasma Secretome. Proteomics. 2019 Aug;19(15):e1900008.

13. Dezfouli M, Vickovic S, Iglesias MJ, Nilsson P, Schwenk JM, Ahmadian A. Magnetic bead assisted labeling of antibodies at nanogram scale. Proteomics. 2014;14(1):14–8.

14. Lin LI-K. A Concordance Correlation Coefficient to Evaluate Reproducibility. Biometrics [Internet]. 1989;45(1):255. Available from: http://www.jstor.org/stable/2532051?origin=crossref

15. McBride G, Bland JM, Altman DG, Lin LI. A proposal for strength-of-agreement criteria for Lin’s Concordance Correlation Coefficient. NIWA Client Rep [Internet]. 2005;45(1):307–10. Available from: http://www.sciencedirect.com/science/article/pii/S0140673686908378%5Cn%3CGo to ISI%3E://A1989U124500022
