# Supplementary material for: Altered levels of CSF proteins in patients with FTD, presymptomatic mutation carriers and non-carriers
Source: Transl Neurodegener. 2020 Jun 23;9:27. doi: 10.1186/s40035-020-00198-y (PMC7310563; doi:10.1186/s40035-020-00198-y)
Supplement: Supplementary file 2 — Additional file 2: Supplementary Figure 1. CSF levels of proteins in Table 2. Statistically significant differences (p-values) are found in Table 2. NC – Non-carriers, PMC – Presymptomatic mutation carriers, PPA – Primary progressive aphasia, bvFTD – Behavioural variant FTD. [file 40035_2020_198_MOESM2_ESM.pdf]

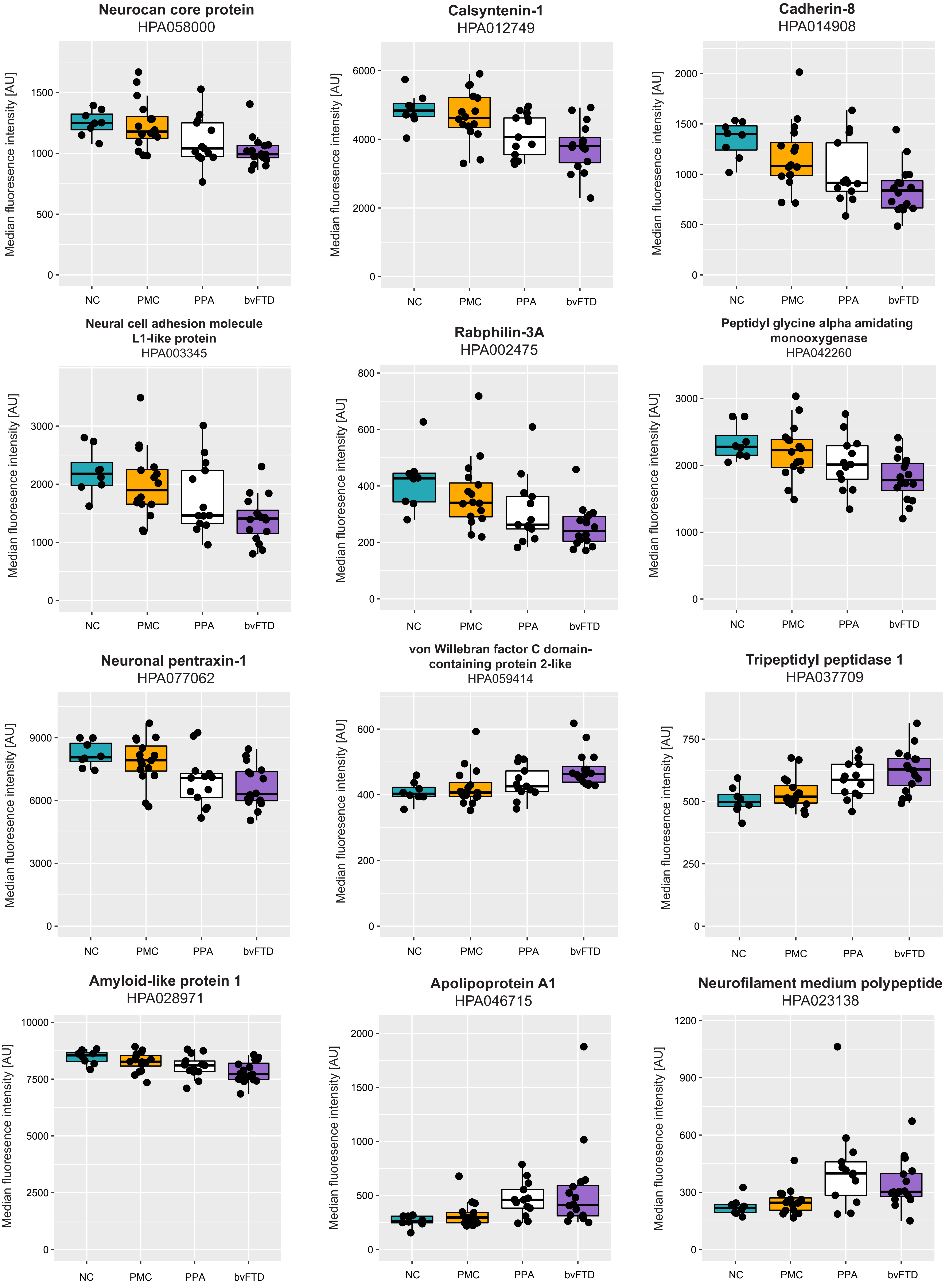
Neurocan core protein
HPA058000
Calsyntenin-1
HPA012749
Cadherin-8
HPA014908
Neural cell adhesion molecule L1-like protein
HPA003345
Rabphilin-3A
HPA002475
Peptidyl glycine alpha amidating monooxygenase
HPA042260
Neuronal pentraxin-1
HPA077062
von Willebran factor C domain-containing protein 2-like
HPA059414
Tripeptidyl peptidase 1
HPA037709
Amyloid-like protein 1
HPA028971
Apolipoprotein A1
HPA046715
Neurofilament medium polypeptide
HPA023138
Median fluoresence intensity [AU]
NC
PMC
PPA
bvFTD

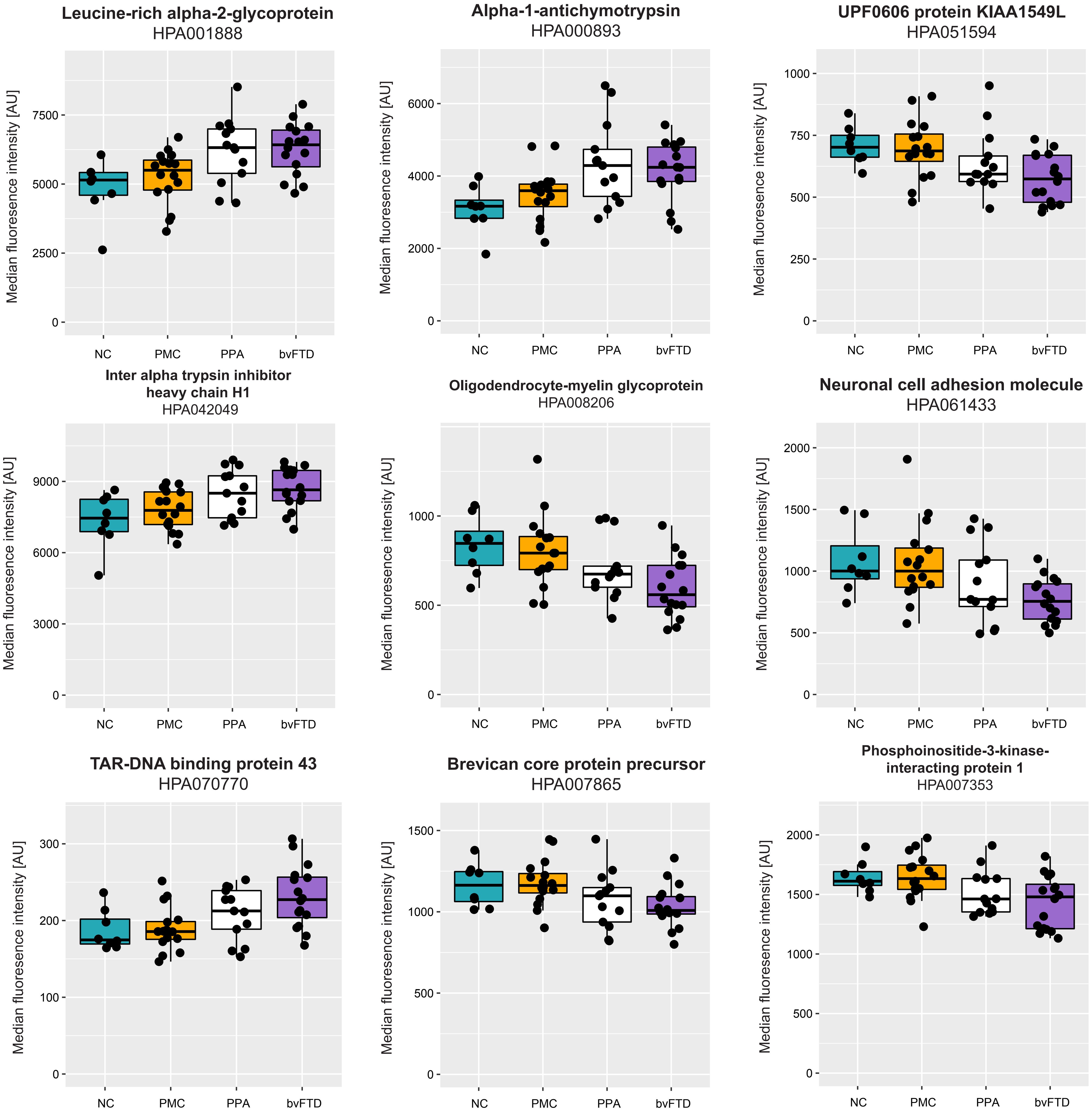
Leucine-rich alpha-2-glycoprotein
HPA001888
Median fluoresence intensity [AU]
0
2500
5000
7500
NC
PMC
PPA
bvFTD
Alpha-1-antichymotrypsin
HPA000893
Median fluoresence intensity [AU]
0
2000
4000
6000
NC
PMC
PPA
bvFTD
UPF0606 protein KIAA1549L
HPA051594
Median fluoresence intensity [AU]
0
250
500
750
1000
NC
PMC
PPA
bvFTD
Inter alpha trypsin inhibitor
heavy chain H1
HPA042049
Median fluoresence intensity [AU]
0
3000
6000
9000
NC
PMC
PPA
bvFTD
Oligodendrocyte-myelin glycoprotein
HPA008206
Median fluoresence intensity [AU]
0
500
1000
NC
PMC
PPA
bvFTD
Neuronal cell adhesion molecule
HPA061433
Median fluoresence intensity [AU]
0
500
1000
1500
2000
NC
PMC
PPA
bvFTD
TAR-DNA binding protein 43
HPA070770
Median fluoresence intensity [AU]
0
100
200
300
NC
PMC
PPA
bvFTD
Brevican core protein precursor
HPA007865
Median fluoresence intensity [AU]
0
500
1000
1500
NC
PMC
PPA
bvFTD
Phosphoinositide-3-kinase-
interacting protein 1
HPA007353
Median fluoresence intensity [AU]
0
500
1000
1500
2000
NC
PMC
PPA
bvFTD
