# Supplementary figures and images for: Altered levels of CSF proteins in patients with FTD, presymptomatic mutation carriers and non-carriers
Source: Transl Neurodegener. 2020 Jun 23;9:27. doi: 10.1186/s40035-020-00198-y (PMC7310563; doi:10.1186/s40035-020-00198-y)

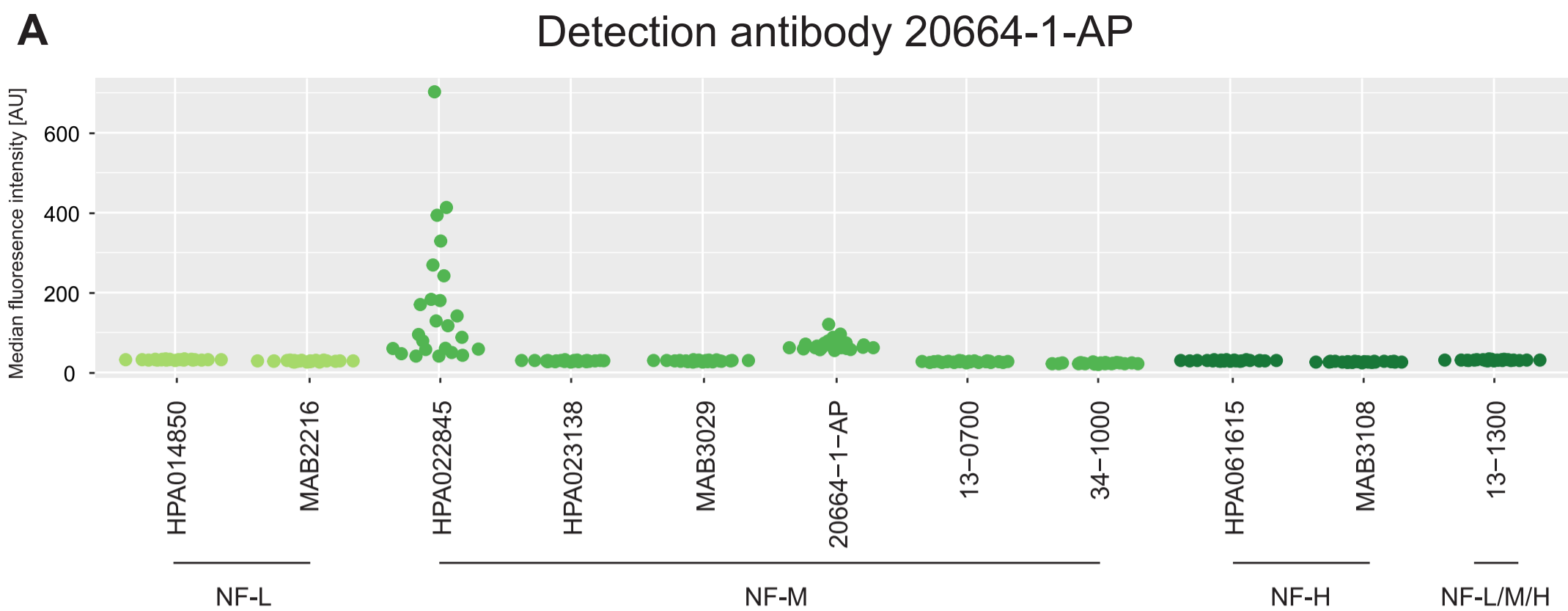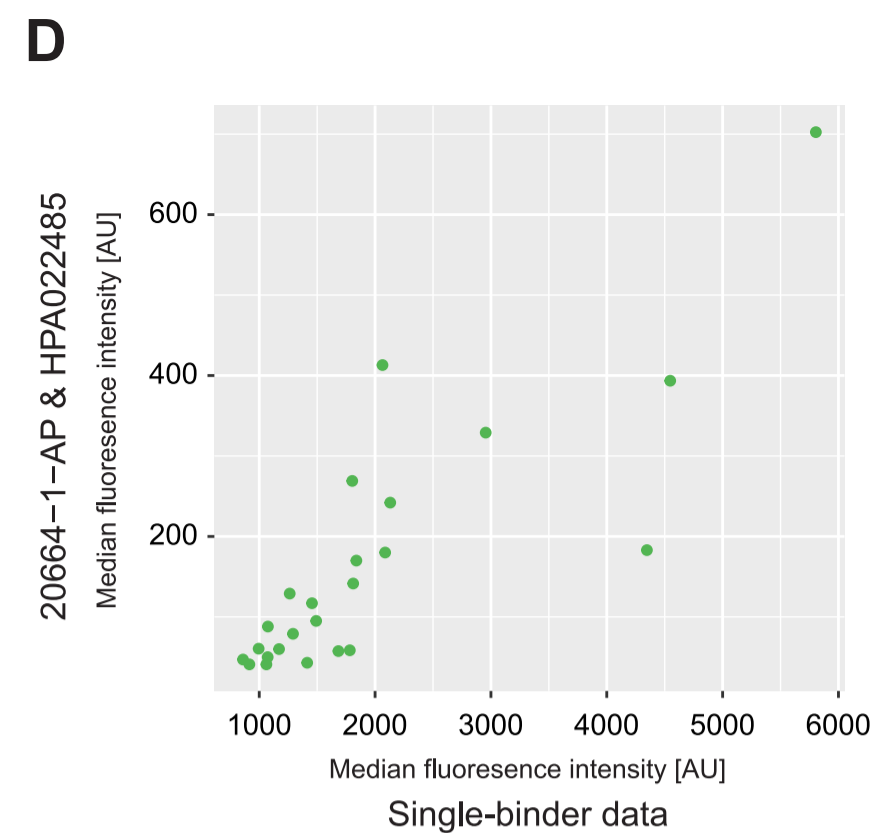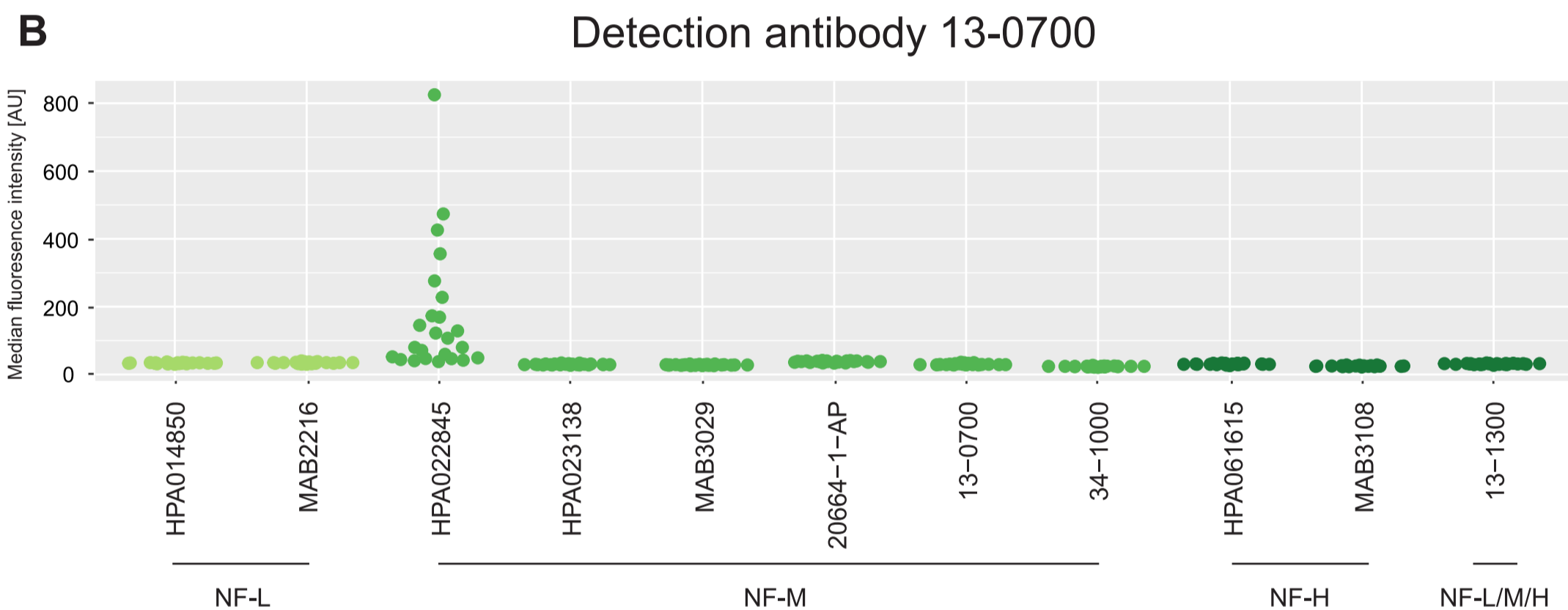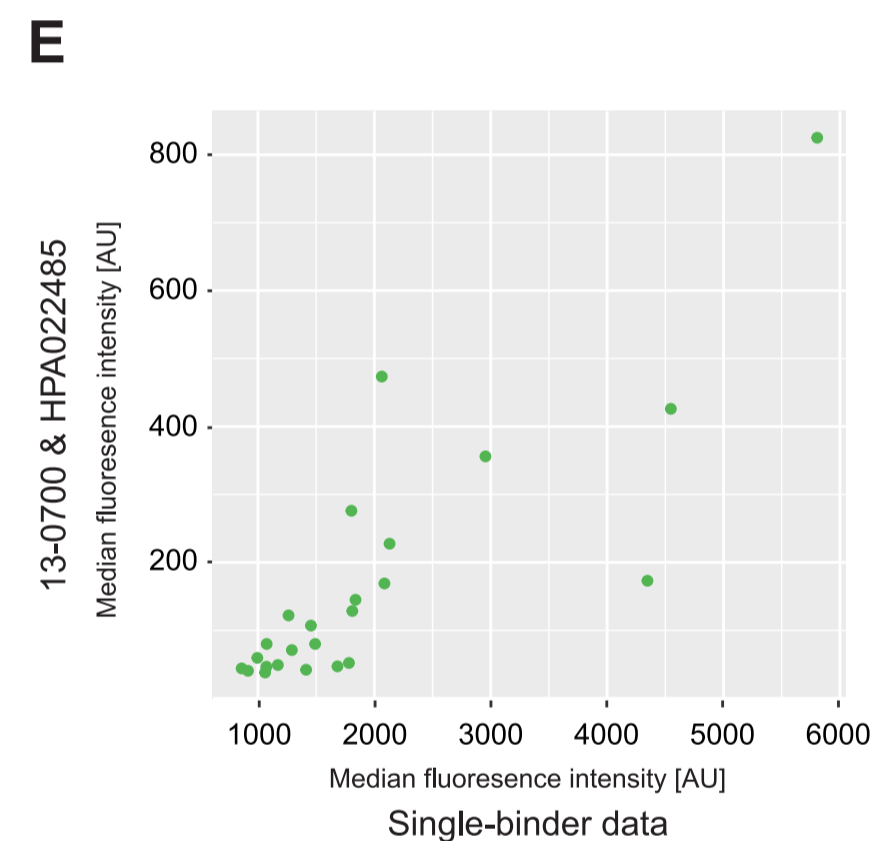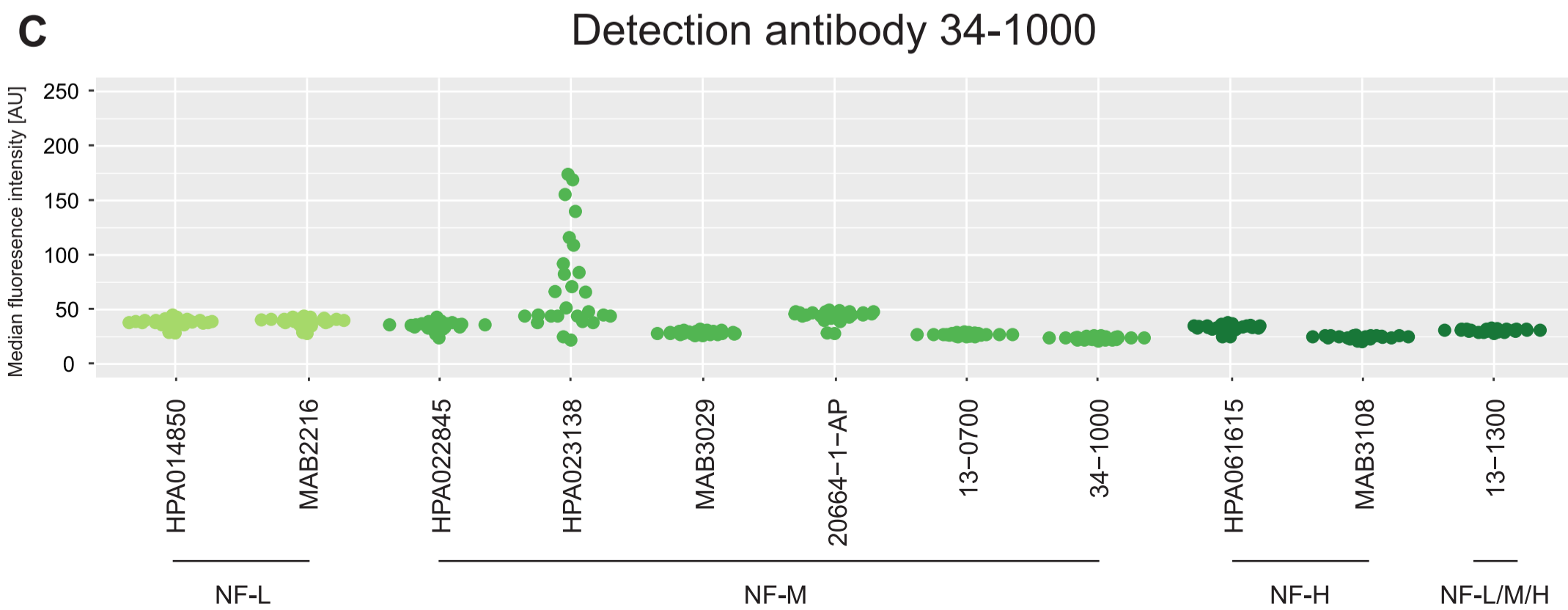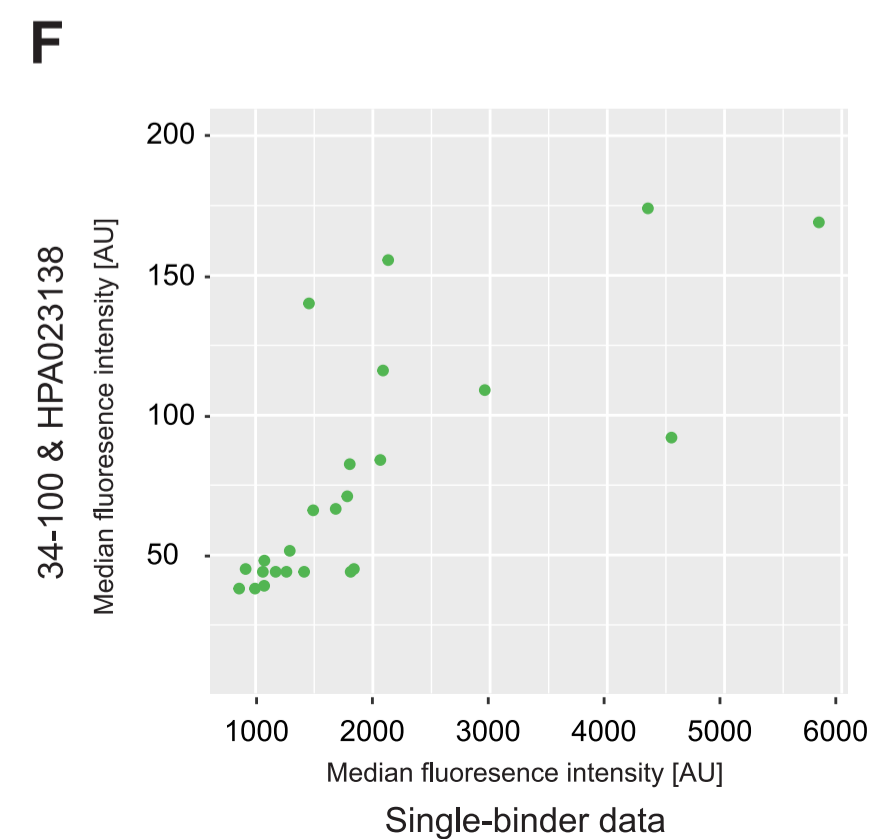

Supplement: Supplementary file 3 — Additional file 3: Supplementary Figure 2. Validation of NF-M antibody binding. (A) Detection of NF-M with HPA022845 as capture antibody and 20,664–1-AP as detection antibody. No cross-reactivity with antibodies targeting NF-L or NF-H was observed. (B) Detection of NF-M with HPA022845 as capture antibody and 13–0700 as detection antibody. No cross-reactivity with antibodies targeting NF-L or NF-H was observed. (C) Detection of NF-M with HPA023138 as capture antibody and 34–1000 as detection antibody. No cross-reactivity with antibodies targeting NF-L or NF-H was observed. (D-F) Comparison between sandwich assay data and single-binder data. Rho(D) = 0.85, p(D) = 1E− 7, rho(E) = 0.86, p(E) = 1E− 7, rho(F) = 0.82, p(F) = 1E− 6. [file 40035_2020_198_MOESM3_ESM.pdf]

**A****HPA022845**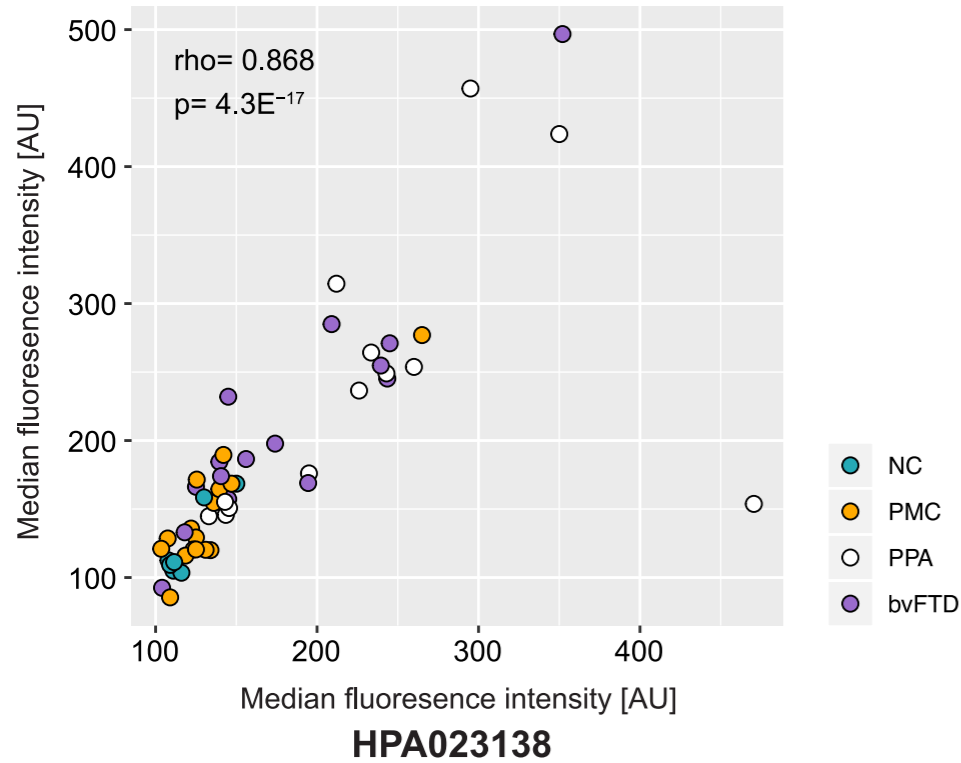**B****20664-1-AP & HPA022845**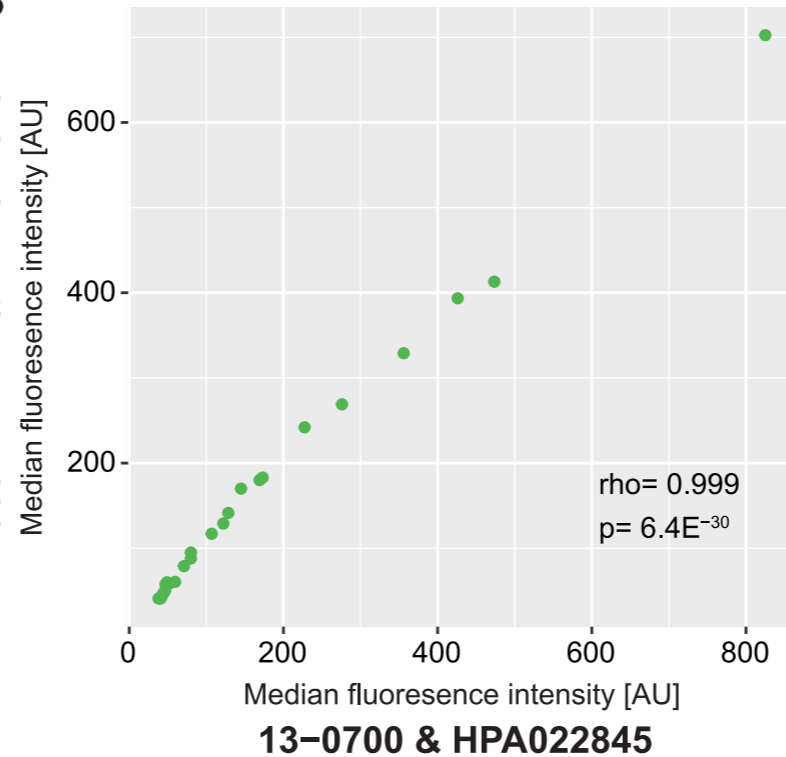**C****20664-1-AP & HPA023138**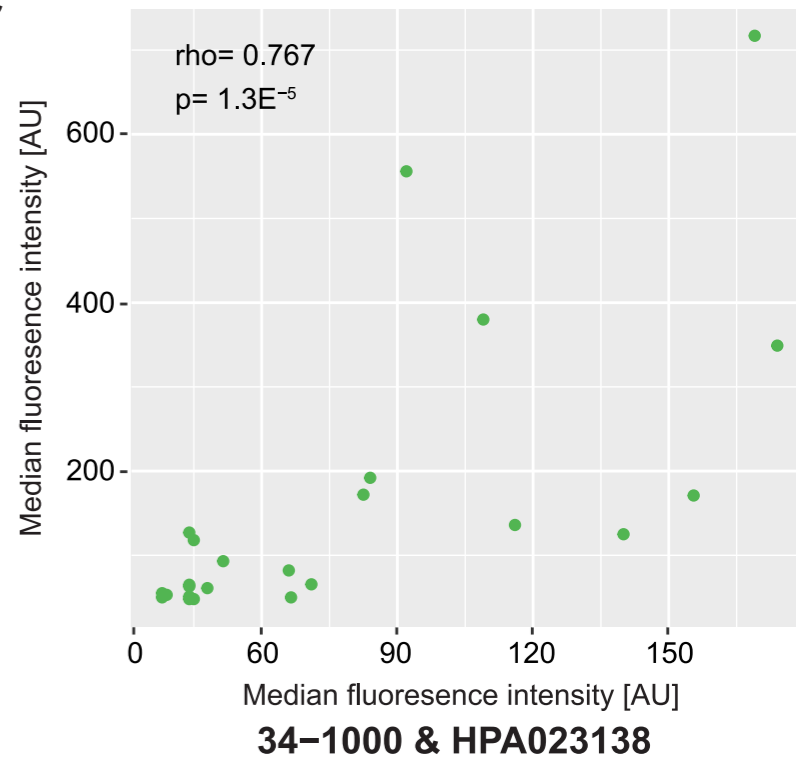

Supplement: Supplementary file 4 — Additional file 4: Supplementary Figure 3. Correlation between NF-M assays. (A) Correlation between HPA022845 and HPA023138 single-binder data. (B) Correlation between HPA022845 sandwich assays. HPA022845 was used as capture antibody together with two different detection antibodies, 20,664–1-AP and 13–0700. (C) Correlation between one HPA22845 sandwich assay and HPA023138 sandwich assay. HPA022845 was used as capture antibody together with 20,664–1-AP as detection antibody, and HPA023138 was used as capture antibody together with 34–1000 as detection antibody. [file 40035_2020_198_MOESM4_ESM.pdf]

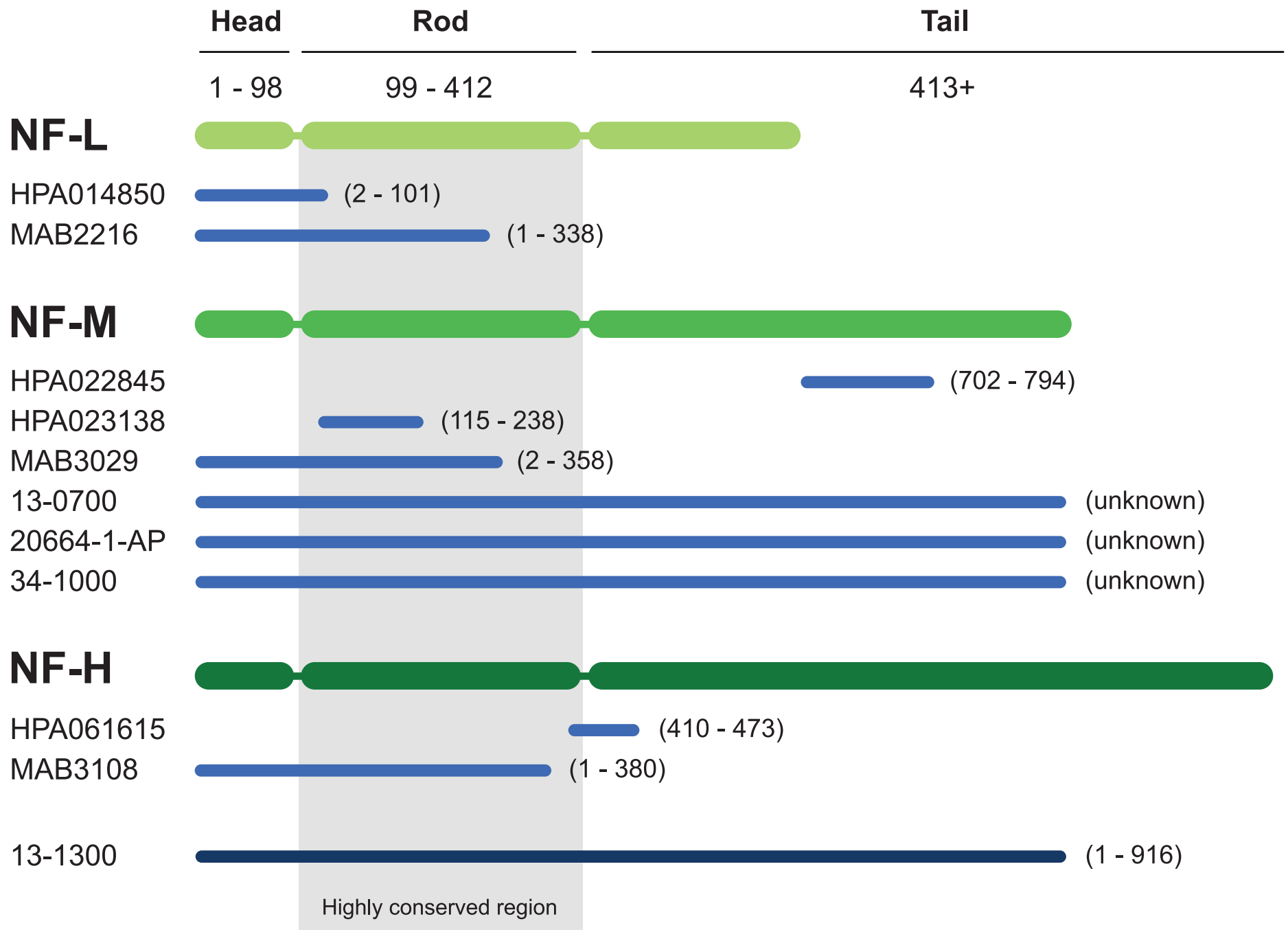

Supplement: Supplementary file 5 — Additional file 5: Supplementary Figure 4. Antibodies used for development of NF-M sandwich assay aligned to the neurofilament amino acid sequences. The neurofilaments are shown in green and the size of the domains (head, rod and tail) are displayed at the top. The highly conserved rod domain is highlighted in grey. Position and length of the amino acid sequences used to generate the antibodies are shown in blue and the exact amino acid positions are given in brackets, if known. Antibody 13–1300 targets all three neurofilaments. The smallest epitope identified for HPA022845 corresponds to amino acids 746–749 [50]. [file 40035_2020_198_MOESM5_ESM.pdf]
